# Deep learning-based automatic tree-level crown detection from high-resolution UAV imagery for aboveground carbon stock estimation

**DOI:** 10.3389/fpls.2026.1958828

**Published:** 2026-09-23

**Authors:** Hancong Fu, Hengqian Zhao, Xiadan Huangfu, Tian Han

**Affiliations:** 1 School of Energy, Environmental and Geomatics Engineering, Anqing Normal University, Anqing, Anhui, China; 2 College of Geoscience and Surveying Engineering, China University of Mining and Technology (Beijing), Beijing, China

**Keywords:** forest AGC stock estimation, high-resolution UAV imagery, individual tree crown detection, local-global feature fusion, YOLOv8-CAFM

## Abstract

Accurate delineation of individual tree crowns is essential for forest resource management and carbon stock estimation. However, traditional manual interpretation is inefficient under complex forest conditions, and existing deep learning-based object detection models remain vulnerable to crown overlap and background interference. To address these limitations, this study proposes an improved YOLOv8-CAFM model. By integrating the local feature extraction capability of convolutional neural networks (CNNs) with the global contextual modeling capability of transformers, the CAFM module enhances the detection of small and partially occluded crowns. Two plantation forests with contrasting canopy densities, dominated by Pinus sylvestris var. mongolica and Pinus tabuliformis in Zhangwu County, Liaoning Province, China, were selected as the study sites. An individual tree crown dataset was manually delineated from high-resolution UAV imagery to support automated crown detection. The proposed model was compared with widely used crown detection models, including Faster R-CNN, YOLOx, and DeepForest. Based on the detected crowns, field measurements, species-specific allometric equations, and carbon content coefficients were further combined to estimate individual-tree aboveground carbon (AGC) stocks. The results showed that YOLOv8-CAFM performed well in both sparse and dense forests, achieving mAP@0.5:0.95 values of 0.736 and 0.609, respectively, and F1-scores of 0.974 and 0.856, outperforming Faster R-CNN, YOLOx, and DeepForest. The extracted crown areas were also highly consistent with the reference data (R² = 0.898 for sparse forests and 0.794 for dense forests), demonstrating strong boundary delineation capability. Furthermore, the estimated forest AGC stocks showed significant correlations with field measurements, with R² values of 0.53 for Pinus sylvestris var. mongolica and 0.60 for Pinus tabuliformis. By jointly modeling local and global features, the proposed method improves individual tree detection, particularly in dense-canopy stand, and provides efficient technical support for accurate forest carbon sink monitoring.

## 1 Introduction

Forests represent a significant component of terrestrial ecosystems, playing a crucial role in mitigating global climate change (De Frenne et al., 2021; Nunes et al., 2020). To achieve sustainable management of forest resources, it is often necessary to conduct a comprehensive forest inventory, including obtaining parameter information such as individual tree height, diameter at breast height (DBH), tree species, crown area, and tree age (Gschwantner et al., 2022). The tree crown is an important site for photosynthesis and transpiration, as well as an important variable for estimating individual tree carbon storage and forest canopy density (Hao et al., 2021; Miraki et al., 2021). Tree crowns form the primary interface through which trees intercept light and exchange carbon and water with the atmosphere. Crown size and spatial arrangement are also shaped by stand structure and competition among neighboring trees. Consequently, reliable individual-crown information is relevant not only to forest inventory but also to the characterization of tree-level structural variation and its relationship with aboveground carbon (AGC) allocation. This information is particularly difficult to recover in closed plantation canopies, where neighboring crowns overlap and exhibit similar spectral and textural characteristics. A key technology of forestry remote sensing is the detection of individual tree crowns, which can help researchers in different fields expand the sampling range for tasks such as forest health monitoring (Cessna et al., 2021), tree species classification (Fassnacht et al., 2016; Ferreira et al., 2019), carbon storage estimation (Sun and Liu, 2020) and pest and disease detection (Duarte et al., 2022).

The traditional method of measuring tree crown mainly relies on field investigation, which calculates the crown area by manually pulling a tape measure to obtain the length of the crown in the east-west and north-south directions. However, this method consumes a lot of manpower and material resources, and is not conducive to the sustainable management and operation of forest resources because of the long survey period, serious impact by the environment, human subjective errors and difficulties in updating data (Gering and May, 1995). The rapid development of remote sensing technology has provided new data acquisition methods for forest resource surveys, and many commercial satellites are capable of capturing ground objects with an area of 1 square meter or even smaller, which is suitable for repeated surveys of forest canopies at large scales and over long-time sequences (Brandt et al., 2020; Lassalle et al., 2022; Tong et al., 2021). However, for specific study areas, it is often difficult to obtain high-resolution image data promptly, making it limited in practical application. Unmanned aerial vehicle (UAV) remote sensing technology has the advantages of convenient use, low cost, fast acquisition of image data and high resolution, which can detect the detailed forest canopy structure at higher spatial resolution, significantly improve the efficiency and accuracy of forest inventory, and at the same time open up a new way to accurately estimate AGC stocks in forests (Huang et al., 2018; Panagiotidis et al., 2017).

Individual tree crown segmentation is a basis for forest AGC stocks estimation, and its accuracy will directly affect the accuracy of the estimation results. The current methods for individual tree recognition based on high-resolution UAV images can be categorized into three main groups: traditional image processing, machine learning (Hartling et al., 2021), and deep learning (Onishi and Ise, 2021; Wu et al., 2020). Traditional image-processing approaches, including multi-scale segmentation (Brandtberg and Walter, 1998), local extreme detection (Van Coillie et al., 2013), pattern matching (Huo and Lindberg, 2020), edge detection (Xia et al., 2022), region growing method (Ma et al., 2020), watershed algorithm (Imangholiloo et al., 2019) and active contouring method (Zhen et al., 2015). Local-maxima and watershed methods are computationally efficient when crown tops and canopy depressions are distinct, but their results are sensitive to smoothing scale, window size, and weak inter-crown depressions, which can cause over or under-segmentation in closed canopies. Edge- and region-based approaches can preserve visible crown contours, but their performance decreases when adjacent crowns have similar spectral characteristics or when shadows interrupt crown edges. Feature-based machine-learning methods can combine spectral and textural predictors, but their transferability depends on the representativeness of the training samples and handcrafted features. These method-specific limitations motivated the increasing use of learned feature representations for crown detection.

In recent years, deep learning technology represented by convolutional neural network (CNN) has made breakthroughs in the field of forest remote sensing (Kattenborn et al., 2021), and has demonstrated significant advantages in tasks such as image recognition and categorization through multi-level feature learning. Deep learning models have been used in studies including individual tree identification (Lassalle et al., 2022), forest fire detection (Kang et al., 2022), plant disease health monitoring (Hu et al., 2022; Yao et al., 2022), and plant phenotype detection (Wang and Su, 2022), all of which combine deep learning algorithms with forest parameter extraction. From the existing literature research results, the deep learning models commonly used for individual tree recognition include Faster R-CNN (Cai et al., 2023; Wang et al., 2019), Mask R-CNN (Hao et al., 2021), YOLO series (Korznikov et al., 2023; Zhang et al., 2022), Retinanet (Roslan et al., 2021), etc., as well as some open-source models specialized in tree crown recognition such as Deepforest (Weinstein et al., 2020), Detectree2 (Ball et al., 2023), and so on. For example, Hao et al. (2021) simultaneously detected crown and tree height parameters in plantation forests by means of a convolutional neural network in a masked region. Puliti and Astrup (2022) proposed an object detection method based on YOLO architecture to recognize trees and achieve good results. Kapil et al. (2024) proposed a self-supervised segmentation method that combines RGB images and thermal imaging images to address issues such as overlapping tree crowns and shadows. Previous studies have already linked crown segmentation to downstream structural estimation. Safonova et al. (2021) combined Mask R-CNN segmentation of olive crowns and shadows with solar geometry to estimate individual-tree height and biovolume. Demissie et al. (2025) further compared segmentation architectures and demonstrated that model choice affects the accuracy efficiency trade off of this workflow. These studies establish that the detection-to-structure sequence is not itself new. However, the behavior of a bounding box detector linked to CHM derived height and species-specific allometry, particularly under contrasting plantation canopy conditions, remains insufficiently characterized.

Despite the progress of deep learning-based crown detection, a specific limitation remains in dense plantation canopies. Conventional YOLOv8 relies predominantly on convolution-based local feature extraction. When adjacent crowns have similar RGB appearance and their boundaries are weakened by overlap, shadow, and background interference, local features alone may be insufficient to represent the broader spatial context needed to distinguish neighboring individuals. This limitation can lead to missed detections, duplicate detections, and inaccurate crown localization, with subsequent effects on crown-derived structural attributes and individual tree AGC estimates. However, the extent to which local–global feature fusion can alleviate this bottleneck across contrasting canopy conditions, and how the remaining crown-detection error affects downstream AGC estimation, has not been sufficiently evaluated. Accordingly, this study addressed three questions: (1) Can the integration of local convolutional features and global contextual attention improve crown detection, particularly in dense and partially occluded canopies? (2) How does the proposed YOLOv8-CAFM model perform relative to conventional YOLOv8, Faster R-CNN, YOLOx, and DeepForest under contrasting canopy conditions? (3) To what extent can UAV-derived crown information support individual tree AGC estimation, and what are the principal limitations and error sources of this multi-step workflow?

## 2 Materials and methods

### 2.1 Study sites

A total of two UAV study areas were selected for this study, both located in Zhangwu County, Fuxin City, Liaoning Province, China (122°53′E, 42°38′N), which were divided into sparse-canopy and dense-canopy stand land, as shown in **Figure 1**. Canopy cover was calculated as the area of the union of all manually delineated crown polygons divided by the total mapped area. The canopy-cover values were 29.1% for the Pinus sylvestris var. mongolica stand and 50.7% for the Pinus tabuliformis stand. Accordingly, the two sites are hereafter referred to as the sparse-canopy and dense-canopy stands, respectively. UAV imagery of the sparse-canopy stand was acquired on 25 July 2022 and covered 15.6 ha, whereas imagery of the dense-canopy stand was acquired on 11 August 2023 and covered 15.1 ha. Both surveys were conducted during the main growing season under clear and low-wind conditions to obtain unobstructed representations of the tree crowns. Zhangwu County belongs to the temperate monsoon continental climate combined with temperate monsoon and continental climate, characterized by obvious seasonal changes throughout the year, with an average annual rainfall of 510 mm, and it is a key county for the construction of the “three norths” protection forests and a pilot county for the afforestation management in the “three norths”. It is also a key demonstration area for “sand prevention and control” in Liaoning Province. Timely and accurate automatic extraction and monitoring of forest land information in the study area can provide services for spatial planning of desertification land and evaluation of ecosystem service value.

**Figure 1:**
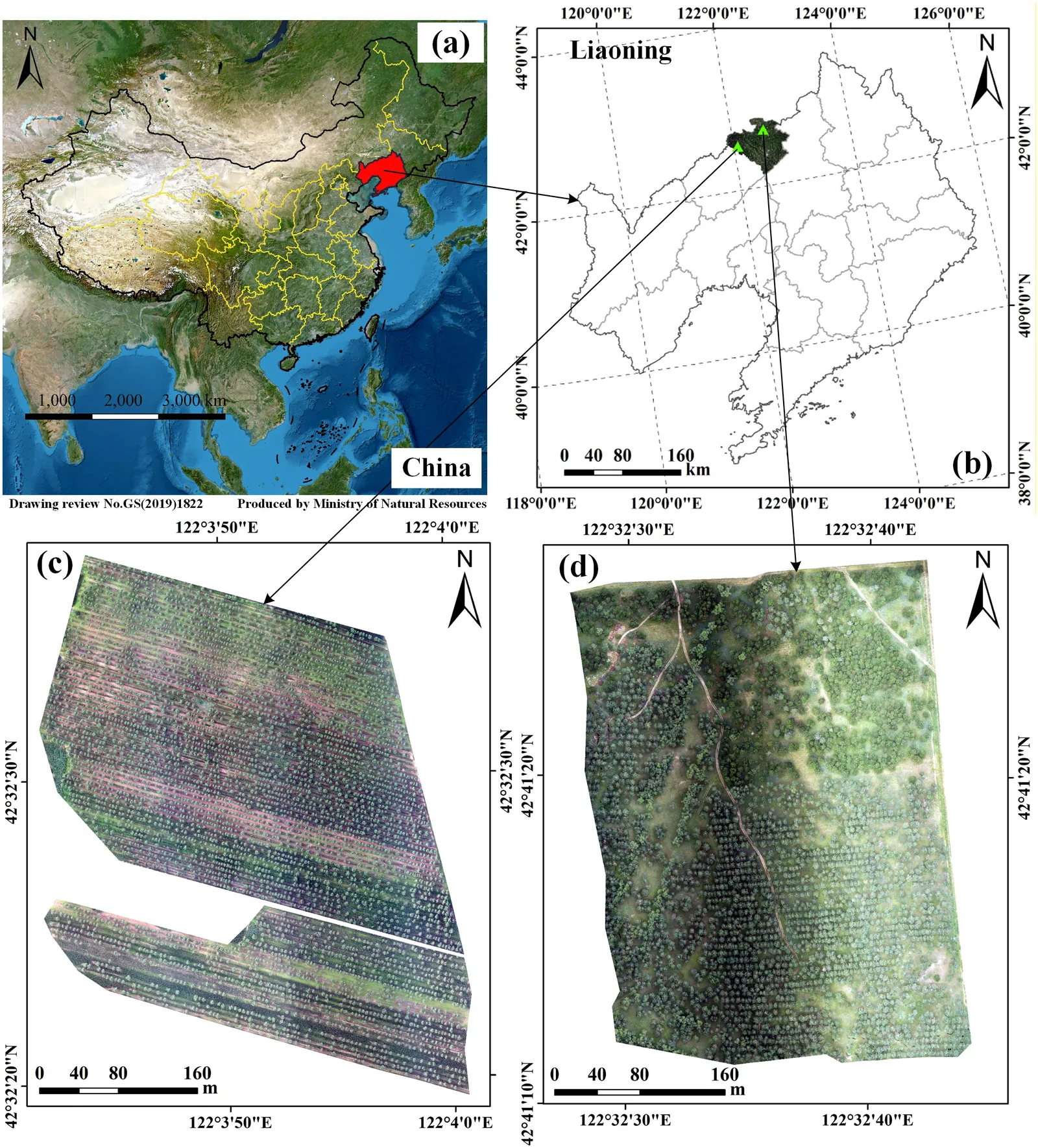
Overview of the research area. **(a)** The red area represents Liaoning Province, China; **(b)** Zhangwu County, Fuxin City, and the locations of the two UAV sample sites; **(c)** UAV DOM of sparse forest; **(d)** UAV DOM of dense forest.

### 2.2 Deep learning model sample labeling and dataset construction

#### 2.2.1 UAV image data acquisition and preprocessing

The UAV image data was obtained using the DJI Phantom 4 RTK platform, and all flight missions were completed under clear weather and low wind conditions to ensure image quality. According to the area and terrain characteristics of the study area, design reasonable flight routes, set the flight altitude to 100m, the main route heading overlap of 80%, and the side overlap of 70%, to meet the accuracy requirements of the later image splicing and 3D reconstruction. The UAV was equipped with an FC6310R digital camera with an image size of 5472×3648 pixels and a pixel size of 2.41 micrometers, containing three spectral channels: red (R), green (G) and blue (B). All collected UAV images were processed and stitched through Pix4dmapper software to generate DOM and Digital Surface Model (DSM). Taking sparse forest as an example, Pix4Demapper combined with 3D Maps processing template was used to process the RGB images of unmanned aerial vehicles, After processing, the average density of point cloud data in the canopy area reached about 872 pt/m^2^, with an elevation accuracy of about 5 cm and a plane accuracy of about 10 cm. The DSM and orthophoto are generated at a resolution of 1 time the project GSD, which is 6.19 centimeters per pixel. In the DSM rasterization process, noise filtering and surface smoothing functions were enabled, with the smoothing type set to sharpness, and the inverse distance weighting method was used for surface interpolation. To obtain the surface, high-density point cloud data is then classified using the automatic machine learning point cloud classification algorithm implemented in Pix4Dmapper. This classifier utilizes the geometric features and RGB attributes of points to classify them into predefined categories, including ground, road surface, tall vegetation, buildings, and man-made structures. Points classified as ground and road surfaces are retained as terrain candidate points, while points classified as vegetation, buildings, and other non-ground categories are excluded. The classification results were manually checked in rayCloud, and the clearly misclassified points were manually corrected before generating the terrain model.

A digital terrain model (DTM, referred to as DEM) was then generated from the corrected terrain points using the automatic class-aware terrain interpolation procedure. The DTM was initially exported at the default resolution of 5 × GSD, approximately 0.31 m, and subsequently aligned with the DSM on the common 0.06 m analysis grid using bilinear resampling. This resampling was performed solely to ensure raster alignment and did not increase the effective spatial information of the original DTM. The canopy height model was calculated as CHM = DSM − DTM and was subsequently used to extract individual-tree height (Mielcarek et al., 2018). Because no independent ground-elevation check points were available for this project, the photogrammetric quality indicators, including the mean reprojection error of 0.092 pixels, were interpreted as measures of internal reconstruction consistency rather than as independent validation of DTM or CHM accuracy.

#### 2.2.2 Labeling of individual tree crown samples

The purpose of tree crown drawing is to evaluate the accuracy of different deep learning algorithms. It has been shown that it is feasible to manually outline tree crowns through UAV imagery (Hao et al., 2021). To ensure that the tree crown annotation results were consistent with the image information, this study was first conducted by a person with rich forestry experience and remote sensing expertise using ArcGIS software to manually outline the edges of individual tree crowns to complete the annotation by visually interpreting the DOM generated from the UAV images, which was then verified by another expert to exclude the potential errors, and the final annotation results were shown in **Figure 2**. It was divided into two study areas, the sparse forest and the dense forest, in which a total of 4965 tree crowns were delineated in the sparse forest and a total of 4144 tree crowns were delineated in the dense forest, The tree crown related parameter information of the two forests was shown in **Table 1**, and the frequency distributions were shown in **Figures 2e, f**. The mean, standard deviation, and coefficient of variation were calculated accordingly. Since different deep learning models have different requirements for the input data format, the data needs to be adjusted according to the model characteristics. This study mainly used the COCO dataset format, firstly, the tree crown vector file was converted to a CSV file which contains the position information of each tree crown box, then it was cropped (512×512 pixels with a fixed overlap rate of 128 pixels) together with the UAV DOM image and the data format was converted to a JSON file. Finally, the data was adjusted according to different modeling requirements and used for the object detection model input. To facilitate the subsequent consolidation of the tree crown detection results, the sparse and dense forests were cropped into four sample plots, A, B, C, and D, respectively, in this study, as shown in **Figures 2a, b**. Among them, sample plots A and C were used for model training and validation (ratio of 7:3), and sample plots B and D were used for model testing.

**Figure 2:**
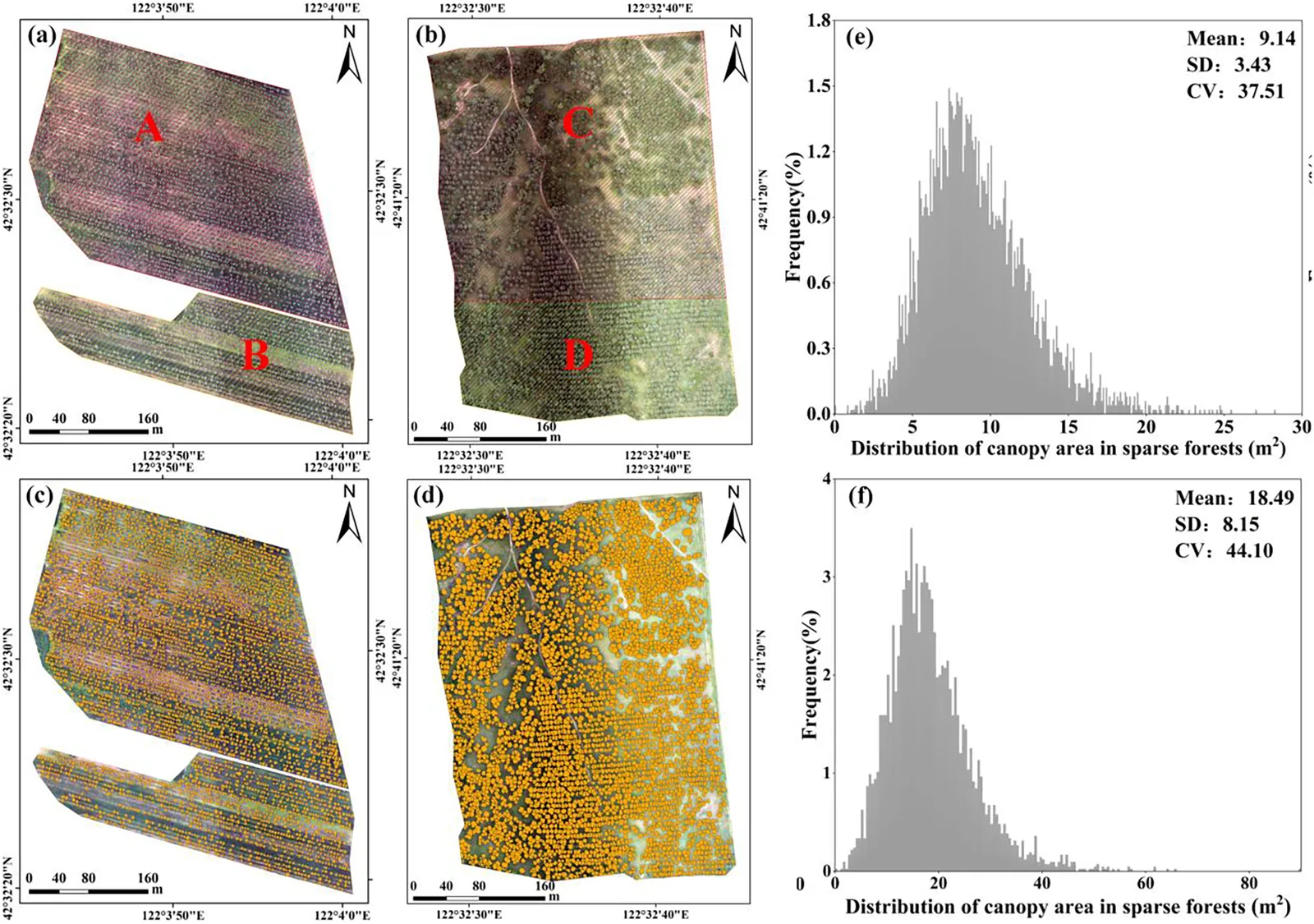
The results of individual tree crown labeling and the frequency distribution map of crown area in forests with different densities in the research area. **(a, c, e)** Sparse forests; **(b, d, f)** Dense forests.

**Table 1:** Statistics of individual tree parameters in different forest densities (m^2^).

Parameters	Sparse forest	Dense forests
Crown area maximum	31.143	89.671
Crown area minimum	0.857	1.399
Crown area mean	9.144	18.489
Crown area standard deviation	3.430	8.153
Canopy cover	29.1%	50.7%

Crown density was calculated from the number of manually delineated crowns divided by the mapped area and should not be interpreted as field-measured stand density because completely occluded trees may not be visible in the UAV imagery. Canopy cover was calculated from the union of manually delineated crown polygons within each study boundary.

#### 2.2.3 Sample dataset cropping and segmentation

Deep learning models have certain limitations on the size of the input image, too large an image may lead to a memory shortage, while too small may lose key information (Sun et al., 2024), so it is necessary to select the appropriate size to crop the image to fit the model input. Also, the image resolution has a significant effect on the tree crown recognition accuracy. It has been shown that higher resolution is not necessarily better (Yin and Wang, 2019), and to facilitate the training and comparison of the models and prevent the pixels of the images input to the models from being too large, in this study, the images resolution in both study areas were resampled to 0.06 m and subsequently processed. To ensure that each tree crown was completely detected in at least one cropped image, both UAV high-resolution images of the two study areas were cropped to a size of 512×512 pixels, and a fixed overlap rate of 25% (128 pixels) was set to facilitate the subsequent merging process of the tree crown detection results, as shown in **Figure 3**. The number of images in the cropped training, validation, and test sets is shown in **Table 2**. The training set and validation set were randomly divided in a 7:3 ratio after pruning, and the test set was not used in model training.

**Figure 3:**
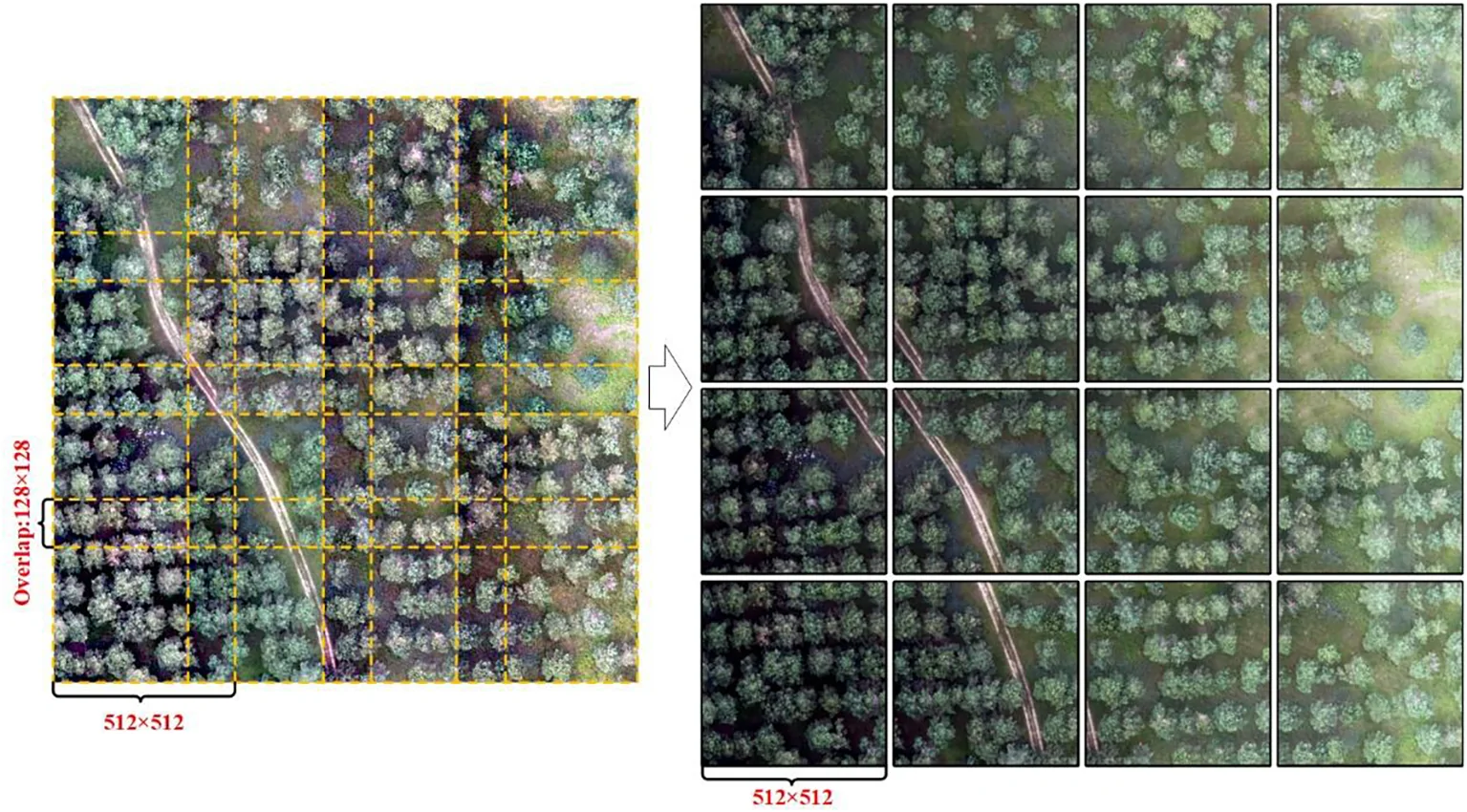
Schematic diagram of the sample cropping method for high-resolution UAV images.

**Table 2:** Statistical results of the images divided into forest datasets with different densities.

Forest	Total number	Train set	Validation set	Testing set
Sparse forest	468	214	92	162
Dense forest	336	157	67	112

#### 2.2.4 Field survey data

In parallel with the UAV image acquisition, field data acquisition was carried out, where four sample plots were accurately measured in each of the two study areas, including 47 trees in the sparse forest and 59 trees in the dense forest, to obtain the species, height and DBH of each tree, and annotated the DOM image using GPS coordinates and expert knowledge, as shown in **Figure 4**. Using a fixed random seed, 24 P. sylvestris var. mongolica trees and 30 P. tabuliformis trees were assigned to the calibration datasets, whereas the remaining 23 and 29 trees, respectively, were reserved for held-out AGC validation. No Tree ID occurred in both datasets. Using a 1:1 ratio for the sample, 24 Pinus sylvestris var. mongolica and 30 Pinus tabuliformis were used to construct the crown diameter equation, while the remaining 23 Pinus sylvestris var. mongolica and 29 Pinus tabuliformis were used for AGC validation. The AGC stock results for individual trees were obtained based on different tree species’ allometric equations and carbon content calculations. Referring to the “Guidelines for Carbon Sequestration Measurement and Monitoring of Afforestation Projects” released by the State Forestry Administration of China, the allometric equations and carbon content used in this study were shown in **Table 3**, and combined with ground field survey data, the individual tree scale carbon stock was obtained as shown in **Figures 4e-h**, which was taken as the true carbon stock value for subsequent accuracy verification.

**Figure 4:**
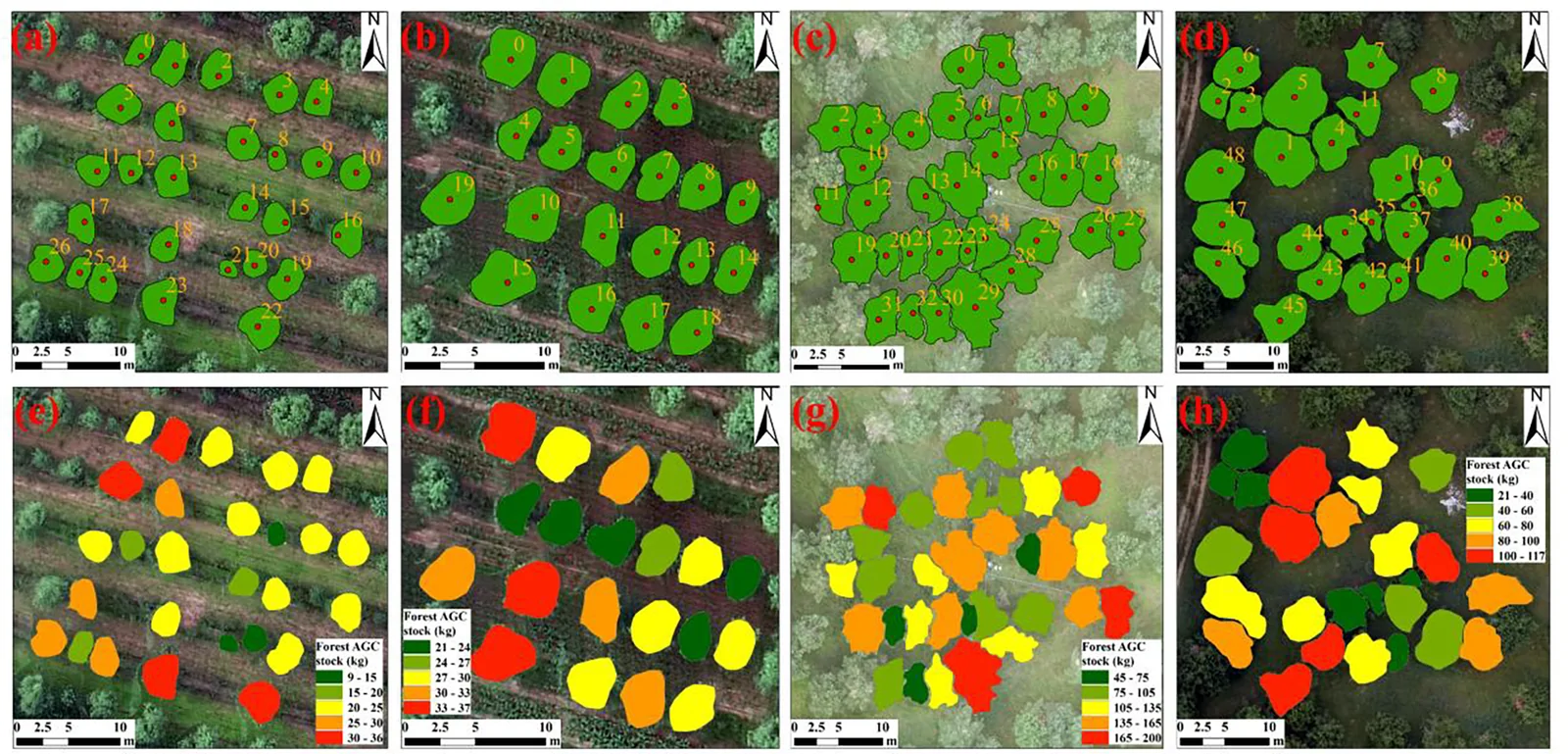
Field collected samples of AGC stocks in individual tree scale forests. **(a, b)** Individual tree sample from a sparse forest containing a total of 47 trees; **(e, f)** Carbon storage of sparse forest individual tree samples; **(c, d)** Individual tree sample from a dense forest containing a total of 59 trees; **(g, h)** Carbon storage of dense forest individual tree samples.

**Table 3:** Allometric equation and carbon content of *Pinus sylvestris* var. *mongolica* and *Pinus tabuliformis*.

Tree species	Allometric equation of aboveground biomass	Carbon content
*Pinus sylvestris* var. *mongolica*	$\text{W}=0.15279({\text{D}}^{2}\text{H}{)}^{0.74238}$	0.5223
*Pinus tabuliformis*	$\text{W}=0.0475({\text{D}}^{2}\text{H}{)}^{0.8539}+0.0017({\text{D}}^{2}\text{H}{)}^{1.1515}+0.0134({\text{D}}^{2}\text{H}{\mathrm{);}}^{0.8099}$	0.5184

### 2.3 Methods

The overall technical framework of this study was shown in **Figure 5**. Based on the YOLOv8-CAFM model, individual tree crowns were segmented in forests with different densities, and individual tree height information was obtained on this basis. Combined with field survey data, different tree species’ allometric equations, and carbon content, individual tree scale forest AGC stock was estimated. The main content includes: (1) Information collection for individual tree field plot investigation; (2) UAV data acquisition and processing; (3) Production and sample partitioning of forest datasets with different densities; (4) Training and accuracy evaluation of different deep learning object detection models; (5) Individual tree scale carbon storage estimation.

**Figure 5:**
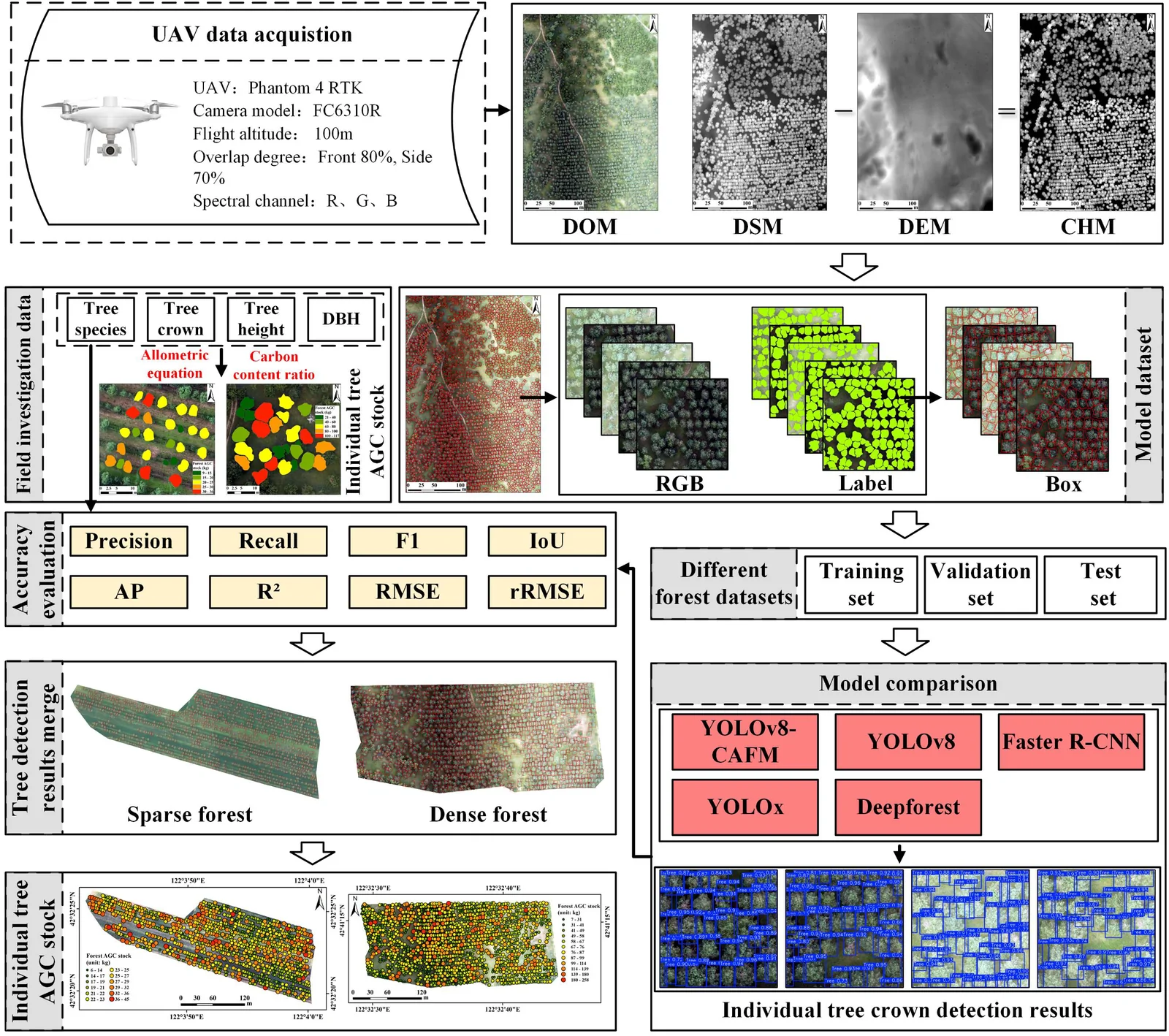
Technical roadmap for individual tree segmentation and carbon stock estimation from high-resolution UAV images based on deep learning.

#### 2.3.1 YOLOv8 model

YOLOv8 was selected as the baseline detector because of its favorable balance between detection accuracy and computational efficiency (Terven et al., 2023). As shown in **Figure 6**, its architecture comprises a backbone, a multi-scale feature fusion network, and a decoupled detection head. The backbone employs CBS, C2f, and SPPF modules to extract hierarchical features; the feature fusion network integrates features across different scales; and the detection head performs object classification and bounding-box regression. In the original C2f blocks, crown representations are generated predominantly through convolutional operations. Although repeated convolutions and feature-pyramid fusion enlarge the effective receptive field, the interaction of spatially distant or contextually related crown features remains indirect. We therefore treated limited explicit long-range feature interaction as a potential bottleneck when local boundaries were weak or adjacent crowns had similar appearance.

**Figure 6:**
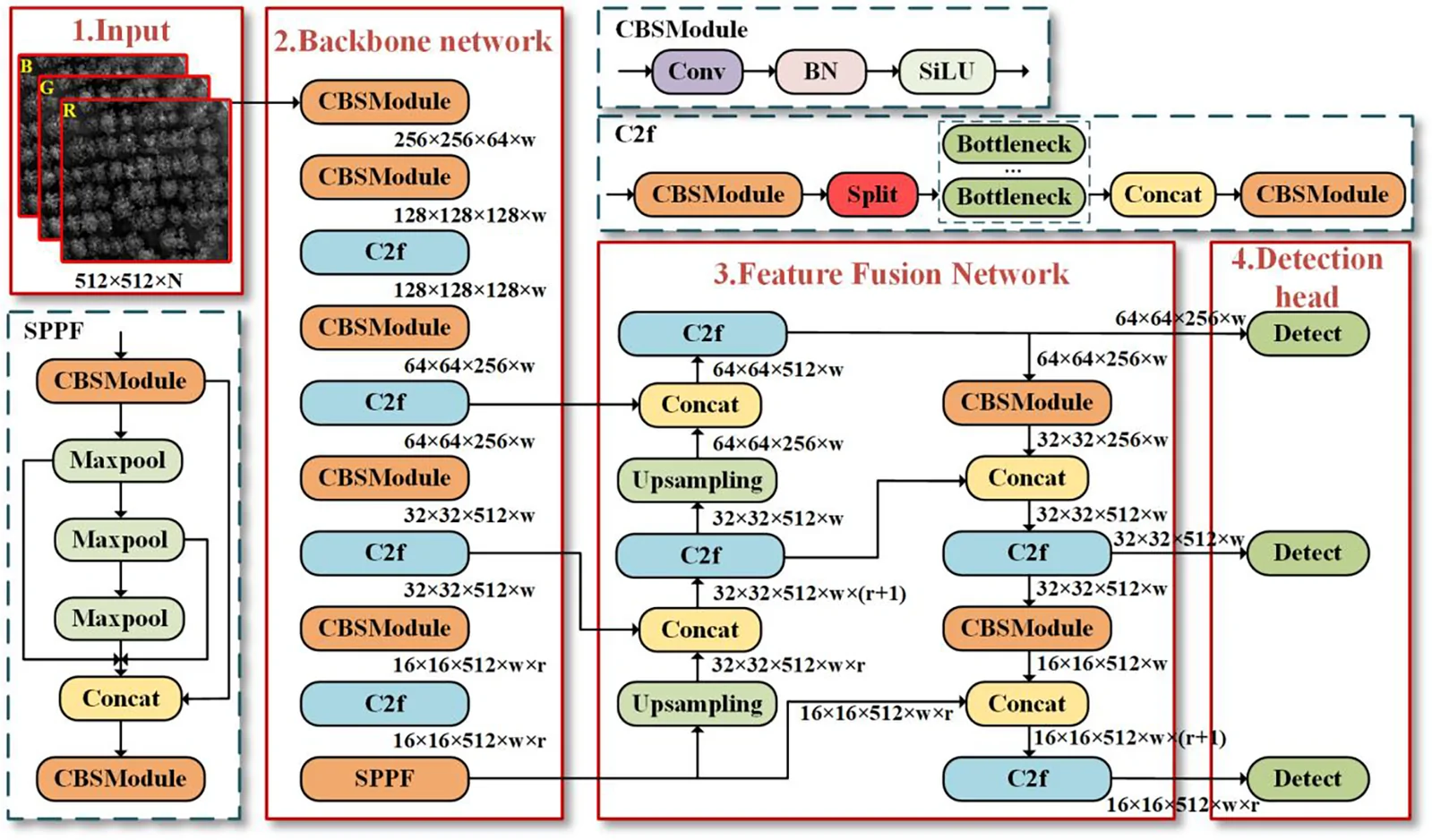
Overall structure of the YOLOv8 individual tree crown object detection network model.

#### 2.3.2 Integration of CAFM into YOLOv8

Currently, the main challenges to individual tree crown detection based on YOLOv8 include crown size, color differences, overlapping crowns in dense areas, and interference from complex backgrounds. In order to improve the detection performance of YOLOv8, this section improves the backbone network by combining CNN and Attention Fusion Module (AFM) (CAFM) on the basis of the original network structure. The CAFM was originally introduced by Hu et al. (2024) to combine convolution-based local feature extraction with attention-based global-context modeling (Hu et al., 2024). Here, we integrated this existing module into the YOLOv8 C2f structure to form C2f-CAFM and evaluated the resulting configuration for individual-tree crown bounding-box detection in high-resolution UAV imagery. Specifically, CAFM was applied to the intermediate feature representation within the C2f block, while the overall YOLOv8 backbone, feature-fusion network, and decoupled detection head followed the baseline architecture.

By improving the C2f module to C2f-CAFM and fully leveraging the advantages of CNN and transformer, the local feature capture capability of CNN and the global feature extraction capability of transformer were utilized to model the local and global features of UAV images, significantly improving the detection performance of small tree crown and occlusion objects in the images (**Figure 7**). At the local scale, the channel dimension was adjusted through a 1x1 convolution without changing the size of the feature map, and then channel shuffling was performed to promote efficient fusion of cross-channel information. After grouping the input tensor by channel dimension, a depth-separable convolution within the group is performed to achieve channel interaction, and then the outputs of each group along the channel axis are formed to form a new feature representation. Finally, by using a 3×3×3 convolution kernel to simultaneously extract spatial and spectral features, local branches can be represented by the following formula (Equations 1–4):

**Figure 7:**
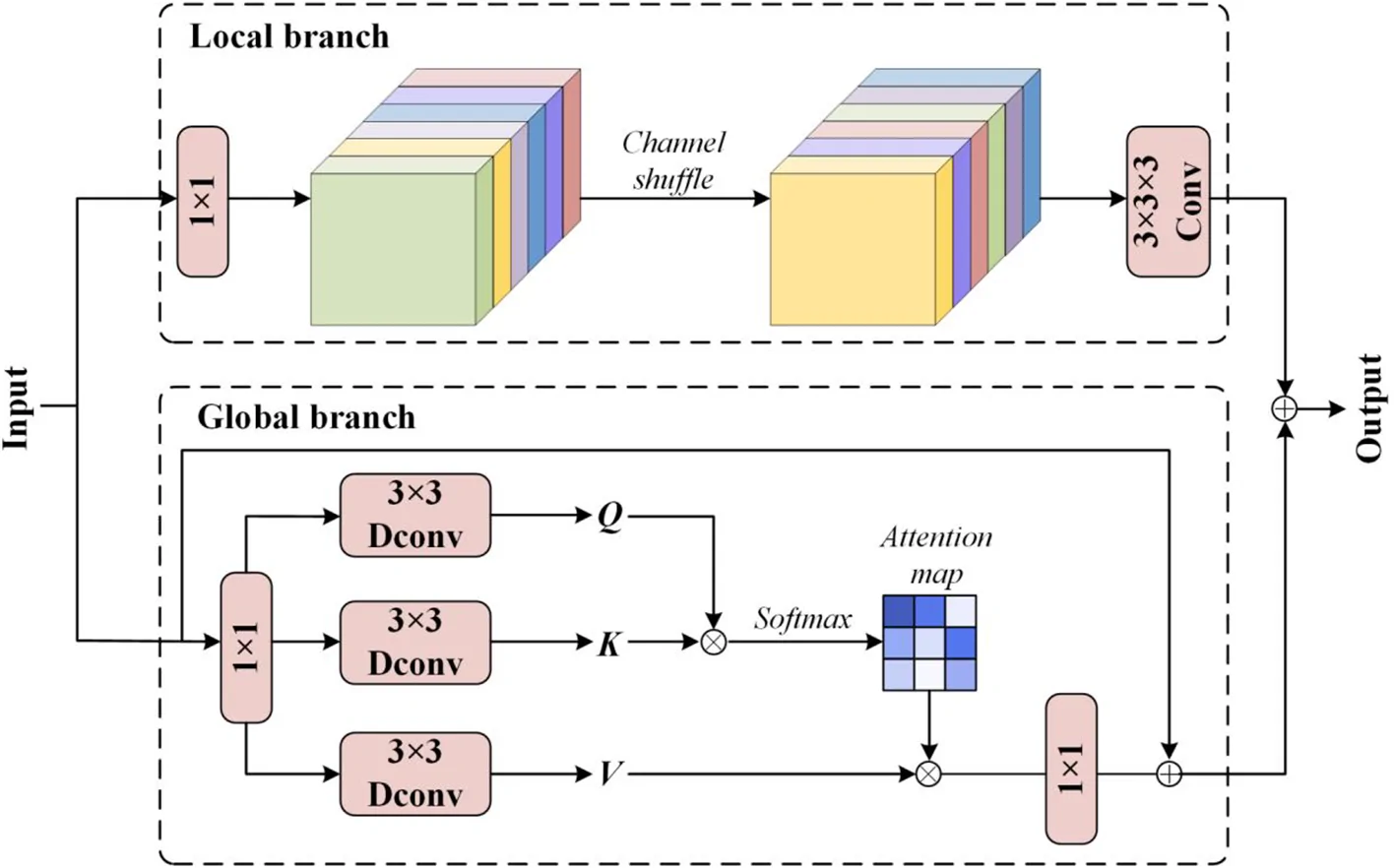
Schematic diagram of the convolution and attention fusion module.

(1) $$F_{conv}=W_{3\times 3\times 3}(CS(W_{1\times 1}(Y)))$$

Where, $F_{conv}$ is the output feature of the local branch, $W_{1\times 1}$ is the 1×1 convolution operation, $W_{3\times 3\times 3}$ is the 3×3×3 convolution operation, CS is the channel shuffling process, and Y is the input feature tensor.

In the global feature processing stage, the Query, Key, and Value vectors were first generated through 1×1 and 3×3 convolutions to obtain three feature tensors with shapes of $\hat{H}\times \hat{W}\times \hat{C}$. Then, Q was reconstructed as $\hat{Q}\in {\mathbb{R}}^{\hat{H}\times \hat{W}\hat{C}}$, K was reconstructed as $\hat{K}\in {\mathbb{R}}^{\hat{C}\times \hat{H}\hat{W}}$, and finally, the $A\in {\mathbb{R}}^{\hat{C}\times \hat{C}}$ attention weight map was calculated using the interaction between $\hat{Q}$ and $\hat{K}$.

The output feature formula $F_{att}$ of the global branch was defined as follows:

(2) $$F_{att}=W_{1\times 1}\mathrm{Attention}(\hat{Q},\hat{K},\hat{V})+Y$$

(3) $$\mathrm{Attention}(\hat{Q},\hat{K},\hat{V})=\hat{V}\mathrm{Softmax}(\hat{K}\hat{Q}/\alpha )$$

where α is a learnable scaling parameter for controlling the size of the multiplication of matrices $\hat{K}$ and $\hat{Q}$.

The fusion module of the convolution and attention mechanism calculates the output as shown in formula (3.13):

(4) $$F_{out}=F_{att}+F_{conv}$$

#### 2.3.3 Experimental environment and parameter settings

All models were trained using the same spatial training, validation, and test partitions. Hyperparameter selection, early stopping, and checkpoint selection were based exclusively on the validation set; the test set was used only for final evaluation. Predictions from all models were converted to the same coordinate system and evaluated using the same reference annotations, IoU definitions, and evaluation code. Because the model families do not share identical loss functions or optimization pipelines, model-specific training recipes were retained where appropriate and are reported explicitly in **Table 4**. Training was performed on Ubuntu 18.04.4 LTS using two NVIDIA GeForce GTX 1080 Ti GPUs, CUDA 11.1, cuDNN 8, PyTorch 1.10.1 (**Table 5**). The reported values represent mean ± standard deviation.

**Table 4:** Summary of model initialization and key training configurations.

Model	Pretrained weights	Optimizer	Initial learning rate	Batch size	Maximum epochs
YOLOv8-n/s/m/l/x	Corresponding Ultralytics YOLOv8 checkpoints pretrained on MS COCO 2017	Auto (SGD selected)	0.01	16	1,500
YOLOv8-CAFM-n/s/m/l/x	Corresponding COCO-pretrained YOLOv8 checkpoints; newly added CAFM layers initialized using the default PyTorch procedure	Auto (SGD selected)	0.01	16	1,500
Faster R-CNN	Torchvision COCO-pretrained Faster R-CNN ResNet-50-FPN checkpoint	SGD	0.005	4	1500
YOLOx	Official yolox_x.pth checkpoint pretrained on MS COCO 2017	SGD with Nesterov momentum	0.0025	16	1500
DeepForest	Official NEON tree-crown release model	SGD	0.001	8	1500

**Table 5:** Experiment platform configuration.

Name	Configuration
Operating system	Ubuntu 18.04.4 LTS
CPU	Intel(R) Core(TM) i7-5930K CPU @ 3.50GHz
GPU	GeForce GTX 1080 Ti * 2
GPU acceleration	CUDA 11.1+cudnn 8
Deep learning framework	Torch 1.10.1

The YOLOv8 baselines and their corresponding YOLOv8-CAFM variants were initialized from the Ultralytics YOLOv8 checkpoints (yolov8n.pt, yolov8s.pt, yolov8m.pt, yolov8l.pt, and yolov8x.pt) pretrained on the MS COCO 2017 dataset. For each YOLOv8-CAFM variant, parameters shared with the corresponding YOLOv8 baseline and having compatible tensor shapes were transferred from the pretrained checkpoint, whereas the newly introduced CAFM layers were randomly initialized using the default parameter-initialization procedure implemented in PyTorch/Ultralytics. Faster R-CNN with a ResNet-50-FPN backbone was initialized from the torchvision checkpoint pretrained on MS COCO. YOLOx was initialized from the official yolox_x.pth checkpoint pretrained on MS COCO 2017. DeepForest was initialized using the official NEON tree-crown release model loaded through use_release in DeepForest version. None of the models was pretrained on imagery from the present study sites; however, the DeepForest release model had been pretrained on external tree-crown annotations from the NEON dataset.

#### 2.3.4 Precision evaluation indicators

To evaluate the performance of different models in individual tree crown detection tasks, Average Precision (AP) and Mean Average Precision (mAP) were selected to evaluate the model results, due to the single object detection of this study, AP and mAP are equivalent. In addition, recall (Buckland and Gey, 1994), precision, and F1 score (Huang et al., 2015) were used to further evaluate the detection performance of the model (Equations 5–8). The recall represents the proportion of correctly identified trees in the model’s prediction results, the precision represents the proportion of correctly identified trees in the sample labels, and the F1 score is based on the harmonic mean of precision and recall, reflecting the overall accuracy of the model.

The Intersection Over Union (IoU) was selected to evaluate the accuracy of individual tree crown masking in different models. It represents the ratio of the overlap area between the predicted box and the true annotated box to the union area, and is used to evaluate the model’s ability to recognize the spatial position of single tree crowns. When the IoU is higher than 50%, it indicates that the crown extraction is correct (Pearse et al., 2020).

(5) $$Precision=\frac{TP}{TP+FP}\times 100\%$$

(6) $$Recall=\frac{TP}{TP+FN}\times 100\%$$

(7) $$F1=\frac{2Precision\times Recall}{Precision+Recall}\times 100\%$$

(8) $$IoU=\frac{area(B_{true}\cap B_{predicted})}{area(B_{true}\cup B_{predicted})}\times 100\%$$

Where *TP* represents the number of correctly detected positive sample tree crowns, *FP* represents the number of incorrectly detected positive sample tree crowns, *FN* represents the number of incorrectly detected negative sample tree crowns, $B_{true}$ represents the true tree crown sample mask, and $B_{predicted}$ represents the predicted tree crown sample mask.

Relying only on precision and recall to evaluate the model performance may be affected by the confidence, resulting in unscientific and limitations. Therefore, mAP are often used to evaluate the recognition performance of the model (Equations 9–10). Where mAP@0.5 refers to the average accuracy when the IoU threshold is 0.5, and mAP@0.5:0.95 refers to the accuracy when the IoU threshold is averaged from 0.5 to 0.95 (with a step size of 0.05), which can reflect the robustness of the model to different overlap rates.

(9) $$AP=\int_{0}^{1}P(R)dR$$

(10) $$mAP=\frac{1}{N}\sum_{c=1}^{N}AP$$

Finally, the real tree crown projection area of the UAV was compared with the tree crown detection area of the optimal model, and three indicators $R^{2}$, RMSE (Hodson, 2022) and rRMSE ( RMSE(%)) were selected to evaluate the accuracy performance of the model’s tree crown area. $R^{2}$ was used for model applicability evaluation, while RMSE and rRMSE were used to quantify the size of the model error (Equations 11–13).

(11) $$R^{2}=1-\frac{\sum_{i=1}^{n}{\left(y_{i}-x_{i}\right)}^{2}}{\sum_{i=1}^{n}{\left(y_{i}-{\bar{y}}_{i}\right)}^{2}}$$

(12) $$RMSE=\sqrt{\frac{1}{n}\sum_{i=1}^{n}{\left(y_{i}-x_{i}\right)}^{2}}$$

(13) $$rRMSE=\frac{RMSE}{{\bar{x}}_{i}}$$

Where n is the number of participants in the accuracy evaluation, $y_{i}$ is the crown area in the predicted result dataset; ${\bar{y}}_{i}$ is the average crown area in the predicted result dataset; $x_{i}$ is the crown area in the reference dataset, and ${\bar{x}}_{i}$ is the average crown area in the reference dataset.

## 3 Result

### 3.1 Training results of tree crown detection models with different densities

**Figure 8** shows the loss curves of YOLOv8 and YOLOv8-CAFM on the test dataset at different model scales, and compares the model performance in different dense forest scenarios. Overall, the loss values of all models showed a significant downward trend and eventually converged, indicating that the prediction error of the models is continuously decreasing. The comparison shows that the loss curves of the five different scale models of YOLOv8 and YOLOv8-CAFM exhibit significant differences during the training process. In this study, under different forest scenarios, the overall loss values of different model scales showed a trend of n>s>m>l>x. Different model scales directly affect the computational complexity and convergence speed of the model, among which the differences between the s, m, l, and x models are relatively small. Therefore, in practical applications, suitable model scales can be selected based on requirements and equipment conditions to balance accuracy and detection speed.

**Figure 8:**
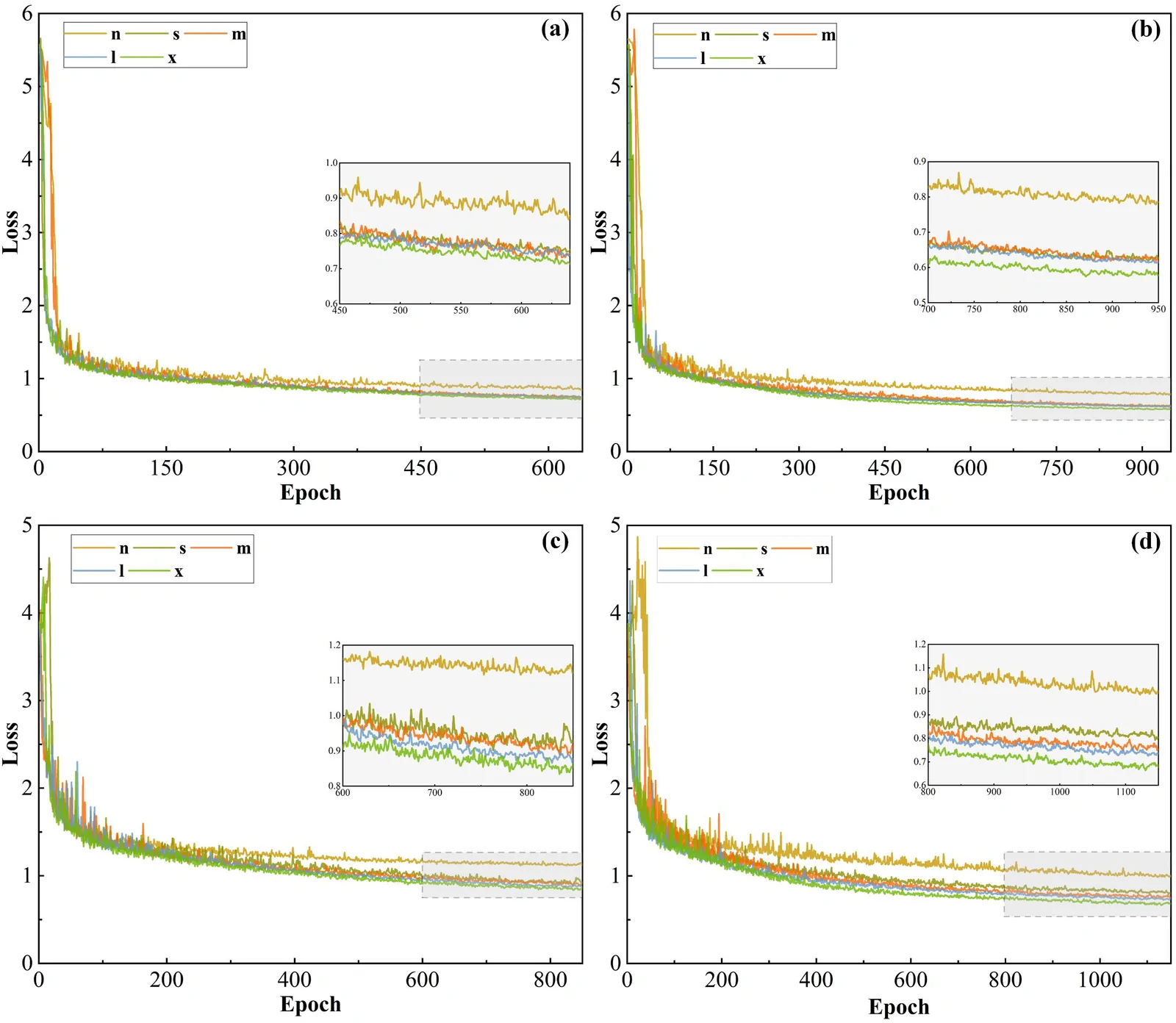
Loss curves of individual tree crown detection at different scales based on YOLOv8 and YOLOv8-CAFM model on the test dataset. **(a)** YOLOv8-sparse forests; **(b)** YOLOv8-CAFM-sparse; **(c)** YOLOv8-dense forests; **(d)** YOLOv8-CAFM-dense forests.

### 3.2 Comparison of tree crown detection accuracy with different densities

Across the five model scales, YOLOv8-CAFM achieved mean absolute gains in mAP@0.50:0.95 of 0.012 and 0.069 over the corresponding YOLOv8 baselines in the sparse- and dense-canopy test sets, respectively. The highest mAP@0.50:0.95 values were 0.736 and 0.609. For mAP@0.50, the mean improvements were 1.3 and 2.1 percentage points, and the maximum values were 98.7% and 88.4%, respectively. For the x-scale model, CAFM increased mAP@0.50:0.95 from 0.721 to 0.736 in the sparse-canopy stand and from 0.541 to 0.609 in the dense-canopy stand (**Figure 9** and **Table 6**). By contrast, mAP@0.50 was 98.7% for both x-scale models in the sparse-canopy stand. Thus, the gain associated with CAFM was larger under dense-canopy conditions and was more apparent when localization was evaluated across stricter IoU thresholds.

**Table 6:** Accuracy statistics of individual tree crown detection based on YOLOv8 and YOLOv8-CAFM model at different scales on the test dataset.

Forest	Model	mAP@0.50	mAP@0.50:0.95	Precision Score=0.5	Recall Score=0.5	F1
Sparse forest	YOLOv8-n	96.2%	0.704	0.949	0.935	0.942
	YOLOv8-s	96.6%	0.713	0.956	0.942	0.949
	YOLOv8-m	96.8%	0.717	0.961	0.947	0.954
	YOLOv8-l	97.3%	0.719	0.965	0.948	0.956
	YOLOv8-x	**98.7%**	**0.721**	**0.975**	**0.971**	**0.973**
	YOLOv8-CAFM-n	97.8%	0.712	0.965	0.954	0.959
	YOLOv8-CAFM-s	98.7%	0.714	0.971	0.962	0.966
	YOLOv8-CAFM-m	98.5%	0.734	0.974	0.967	0.970
	YOLOv8-CAFM-l	98.4%	0.736	0.975	0.968	0.971
	YOLOv8-CAFM-x	**98.7%**	**0.736**	**0.978**	**0.970**	**0.974**
Dense forest	YOLOv8-n	84.6%	0.523	0.872	0.756	0.810
	YOLOv8-s	85.5%	0.534	0.876	0.775	0.822
	YOLOv8-m	85.5%	0.534	0.883	0.777	0.827
	YOLOv8-l	85.6%	0.535	0.886	0.778	0.828
	YOLOv8-x	**86.7%**	**0.541**	**0.895**	**0.805**	**0.848**
	YOLOv8-CAFM-n	87.2%	0.587	0.886	0.796	0.839
	YOLOv8-CAFM-s	87.6%	0.602	0.890	0.797	0.841
	YOLOv8-CAFM-m	87.6%	0.606	0.896	0.802	0.846
	YOLOv8-CAFM-l	87.8%	0.608	0.902	0.810	0.854
	YOLOv8-CAFM-x	**88.4%**	**0.609**	**0.902**	**0.814**	**0.856**

Bold font for optimal accuracy.

**Figure 9:**
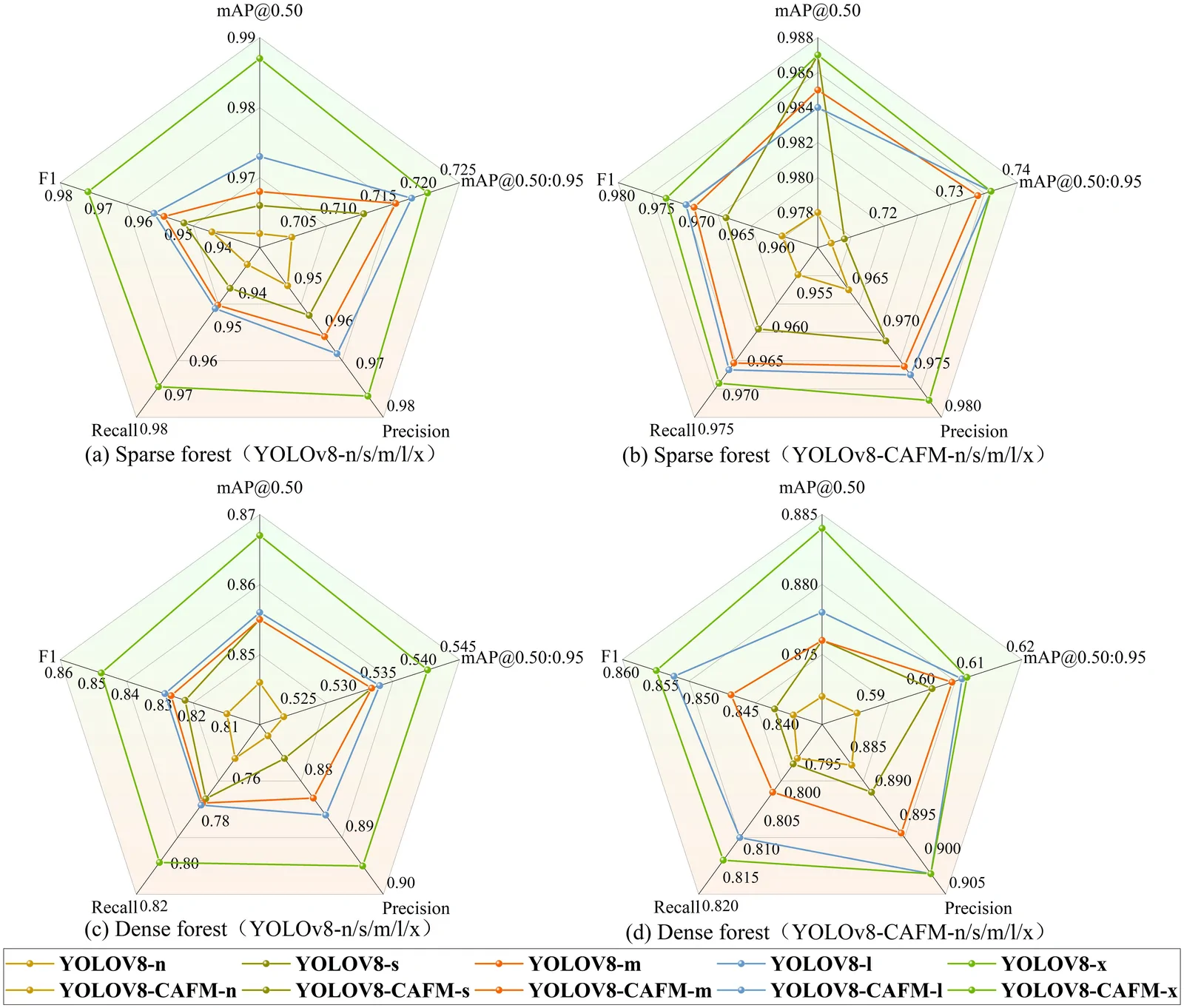
Comparison of individual tree crown detection accuracy based on YOLOv8 and YOLOv8-CAFM model at different scales on the test dataset. **(a)** YOLOv8-sparse forests; **(b)** YOLOv8-CAFM-sparse; **(c)** YOLOv8-dense forests; **(d)** YOLOv8-CAFM-dense forests.

### 3.3 Comparison of tree crown object detection results and accuracy of different models

This section compares the YOLOv8-CAFM model with three commonly used models: Faster R-CNN, YOLOx, and Deepforest. The loss function curves of the three models during training in sparse and dense forests are shown in **Figure 10**. To avoid subjective selection of training length, a predefined early-stopping procedure was used. All principal models were assigned a maximum training budget of 1500 epochs and were evaluated on the same validation set after each epoch. Training was terminated when the improvement of Loss value within 10 consecutive epochs is less than 0.001. The checkpoint achieving the highest validation was retained for final evaluation. Consequently, the actual number of completed epochs differed among models because of differences in convergence rate, whereas the maximum training budget, stopping criterion, validation data, and checkpoint-selection rule were held constant. From the perspective of the global convergence trend, all models exhibit a monotonic decrease in loss function and ultimately reach steady-state equilibrium, verifying the rationality of the experimental setup. However, there are significant differences in the loss curve characteristics among different model architectures: Faster R-CNN and YOLOx models exhibit smooth convergence curves in both sparse and dense forests, while Deepforest shows significantly high-frequency oscillations throughout the training process. It is worth noting that the final loss convergence values of all models in dense forests are systematically higher than those in sparse forests, which may be due to the increased difficulty of model detection caused by tree crown overlap in dense scenes.

**Figure 10:**
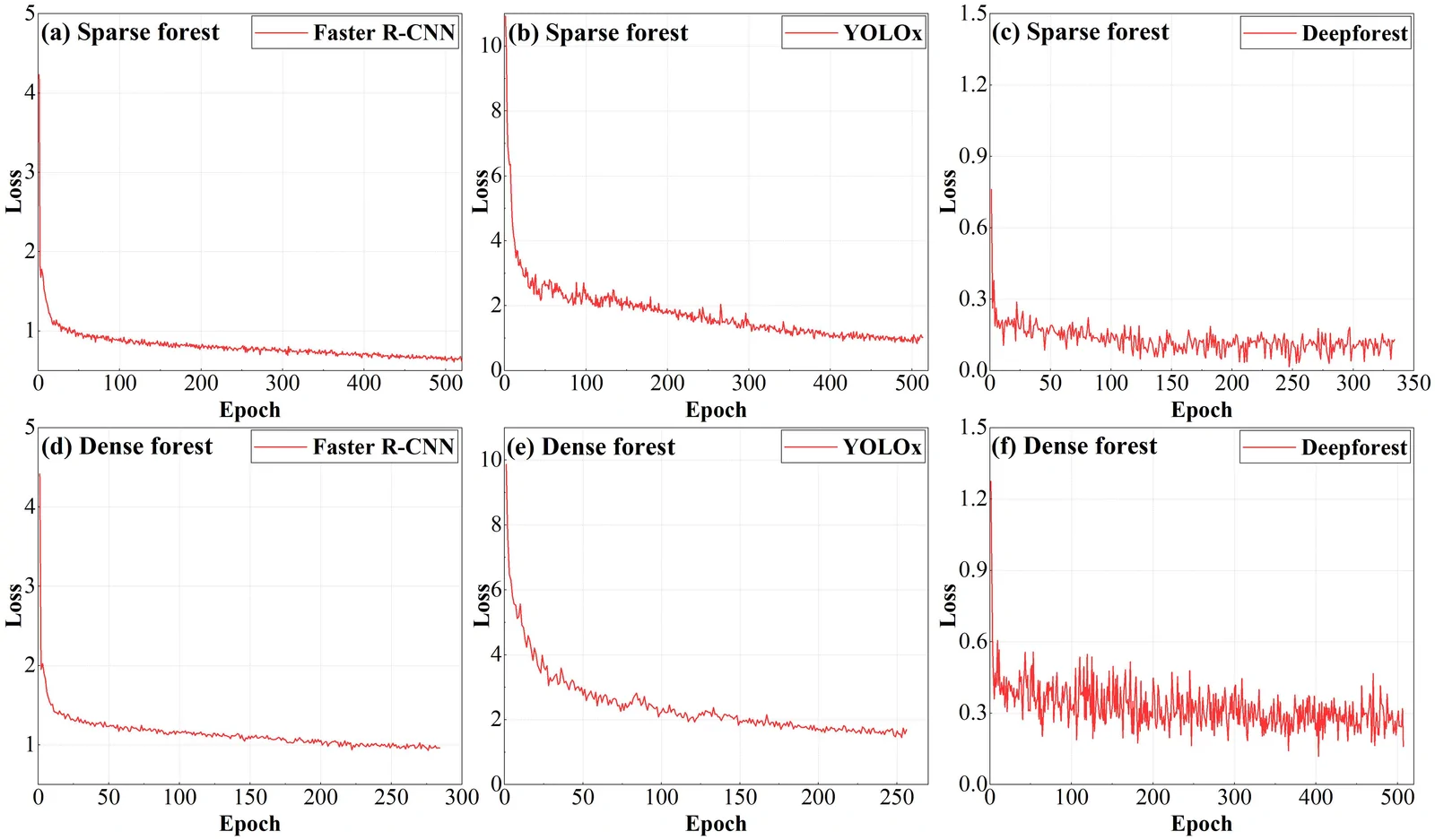
Loss function variation curves of different individual tree detection models. **(a)** Sparse forest-Faster R-CNN; **(b)** Sparse forest-YOLOx; **(c)** Sparse forest-Deepforest; **(d)** Dense forest-Faster R-CNN; **(e)** Dense forest-YOLOx; **(f)** Dense forest-Deepforest.

**Figure 11** and **Table 7** show the comprehensive evaluation results of different individual tree detection models based on the test set in two typical scenarios (sparse forest and dense forest). The experimental results showed that the YOLOv8-CAFM (x) model performs optimally and achieved the best detection performance in both scenarios, followed by YOLOv8, Faster R-CNN, YOLOx, and Deepforest. In sparse forest scenes, all models exhibited high detection accuracy, the mAP@0.50:0.95 indicators were 0.736, 0.721, 0.669, 0.629, and 0.622, respectively, and the F1 values were 0.974, 0.973, 0.949, 0.928, and 0.917, respectively. In dense forest scenes, the detection difficulty significantly increases, resulting in a decrease in the accuracy of different models compared to sparse forest scenes, the mAP@0.50:0.95 indicators were 0.609, 0.541, 0.526, 0.492, and 0.375, respectively, and the F1 values were 0.856, 0.848, 0.815, 0.785, and 0.710, respectively. Despite the decrease in accuracy, the YOLOv8-CAFM(x) model maintains a high detection performance.

**Figure 11:**
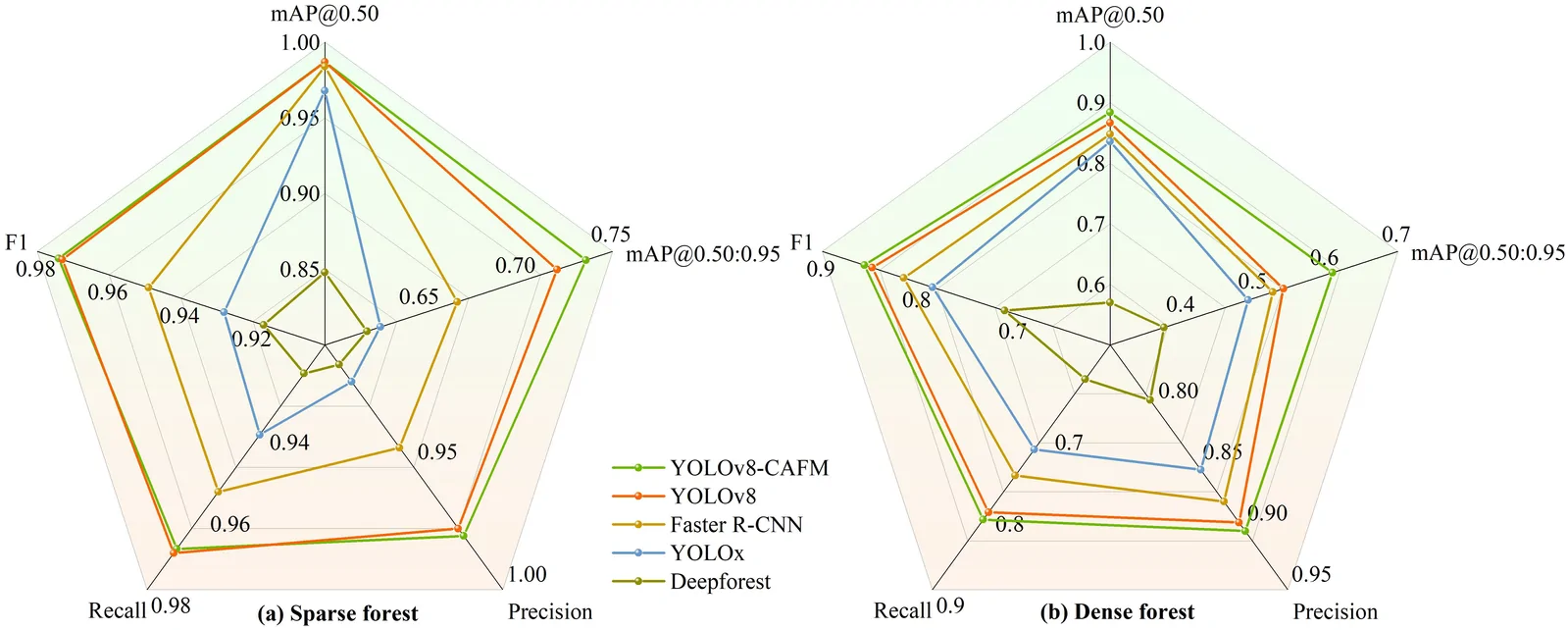
Evaluation accuracy of different individual tree crown detection models on the test dataset **(a)** Sparse forest; **(b)** Dense forest.

**Table 7:** Evaluation accuracy of different individual tree detection models on the test dataset.

Forest	Model	mAP@ 0.50	mAP@0.50:0.95	Precision Score=0.5	Recall Score=0.5	F1
Sparse forest	YOLOv8-CAFM(x)	**98.7%**	**0.736**	**0.978**	0.970	**0.974**
	YOLOv8(x)	98.7%	0.721	0.975	**0.971**	0.973
	Faster R-CNN	98.4%	0.669	0.942	0.956	0.949
	YOLOx	96.8%	0.629	0.915	0.942	0.928
	Deepforest	84.8%	0.622	0.908	0.927	0.917
Dense forest	YOLOv8-CAFM(x)	**88.4**%	**0.609**	**0.902**	**0.814**	**0.856**
	YOLOv8(x)	86.7%	0.541	0.895	0.805	0.848
	Faster R-CNN	84.8%	0.526	0.878	0.760	0.815
	YOLOx	83.6%	0.492	0.852	0.728	0.785
	Deepforest	57.0%	0.375	0.795	0.642	0.710

Bold font for optimal accuracy.

The qualitative examples in **Figure 12** showed that differences among models were limited for isolated crowns with clear boundaries. In dense-canopy examples, YOLOv8-CAFM-x produced fewer missed crowns and fewer false positives associated with shadows or background objects than the comparison models. The remaining errors were concentrated where neighboring crowns strongly overlapped or had indistinct boundaries. These visual patterns were consistent with the larger quantitative gain observed in the dense-canopy test set.

**Figure 12:**
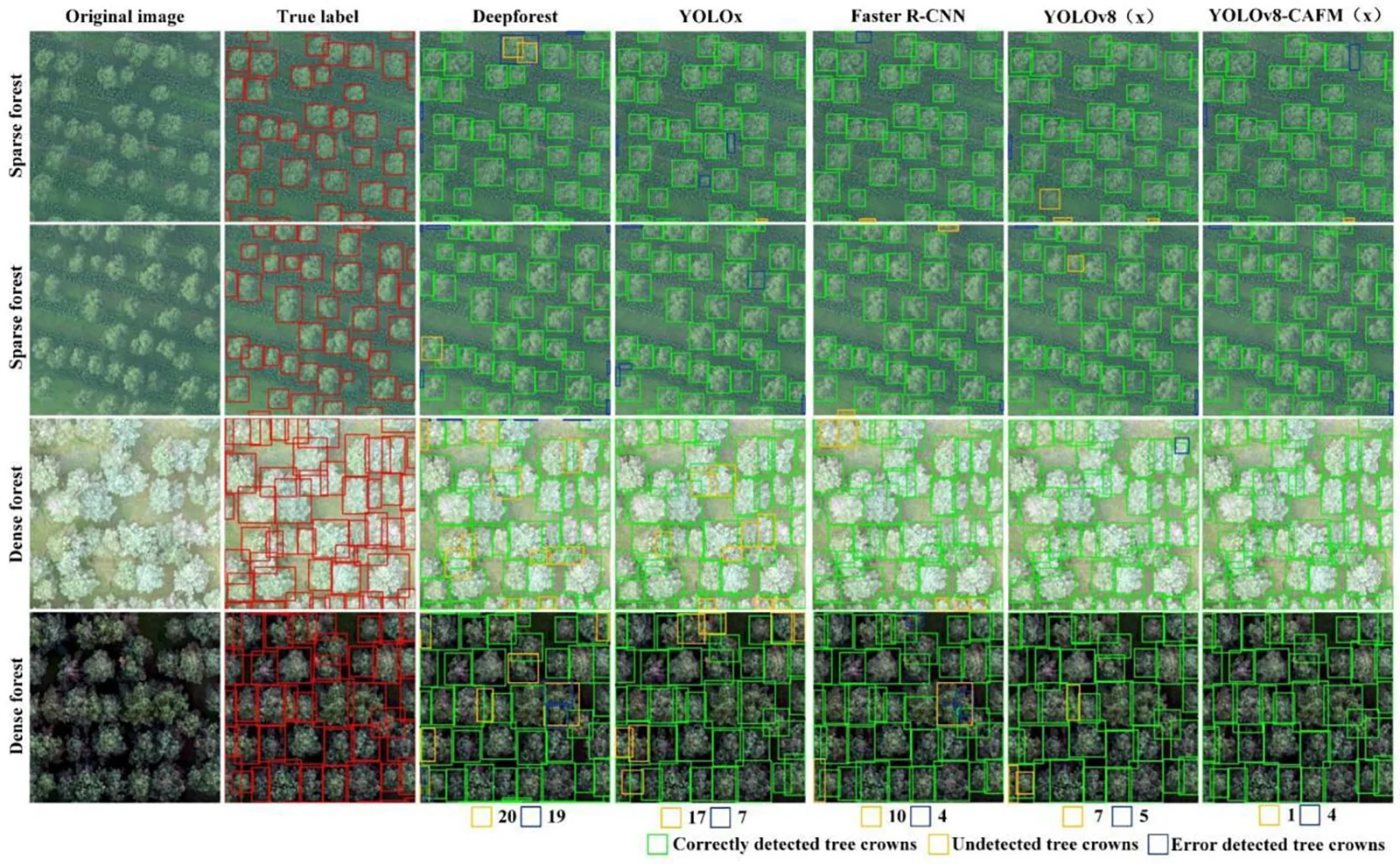
Case study of individual tree crown detection results from different models.

### 3.4 Individual tree crown object detection results merge and area evaluation

A remote sensing image object detection result merging algorithm based on spatial position constraints and grouping optimization was proposed. The specific steps were as follows. Firstly, based on spatial topological relationships, association rules for detection boxes were constructed. For any two detection boxes, if the center point of one detection box fell within the range of the other detection box, these two detection boxes were divided into the same group. By traversing all detection boxes, they were finally divided into several groups, where each group represented an independent tree crown object. To optimize the merging effect, this section calculates the extreme values of X_min_, Y_min_, X_max_, and Y_max_ of all detection boxes within the group, and extracts the minimum bounding rectangle of each detection box as the new detection box, which not only preserves the geometric integrity of the target, but also significantly reduces the redundancy of detection boxes in overlapping areas. Considering that there are usually many individual trees in remote sensing images, directly using global traversal to compare detection boxes will lead to a sharp increase in computational complexity. Therefore, this study also introduces an optimization strategy based on gridded spatial indexing, dividing the research area into regular grids and only comparing and analyzing detection boxes within the same or adjacent grids, significantly reducing unnecessary computational complexity.

The individual tree crown detection results of YOLOv8-CAFM (x), YOLOv8 (x), Faster R-CNN, YOLOx, and Deepforest were merged using the above merging algorithm, and the extracted crown numbers were statistically analyzed. In sparse forests, the number of individual tree crowns in the above five models is 1046, 1037, 1017, 977, and 934, respectively. The deviations from the actual sample size are 19, 28, 48, 88, and 131, respectively. In dense forests, the detection performance of all models has decreased. The statistical numbers of individual tree crowns are 1446, 1383, 1351, 1229, and 1217, respectively. The deviations from the actual sample numbers are 61, 124, 156, 278, and 290, respectively. In both scenarios, the YOLOv8-CAFM(x) model exhibited the highest detection accuracy, which is consistent with the aforementioned accuracy evaluations (mAP and F1 values).

This section also presents the merged results of the YOLOv8-CAFM (x) model on different forest test sets (**Figures 13**, **14**). From the figure, it can be seen that although there are problems such as tree crown obstruction and uneven lighting, the model can clearly identify the boundaries of individual tree crowns and effectively avoid background interference. It can complete the tree crown extraction task well and meet practical investigation needs.

**Figure 13:**
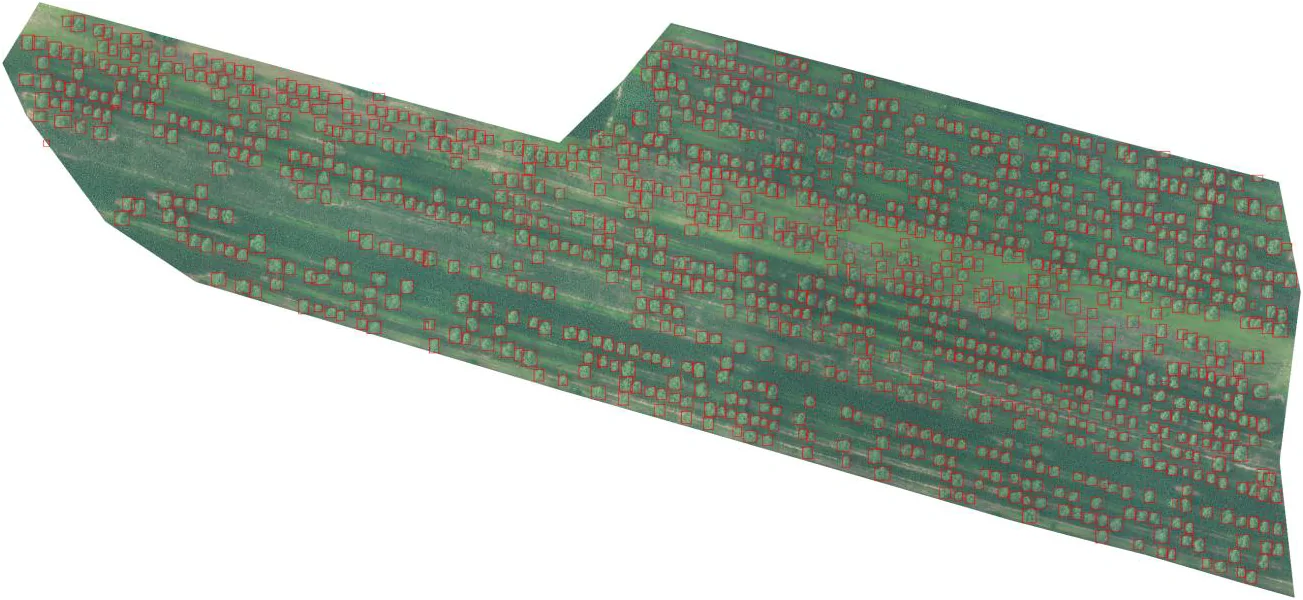
YOLOv8-CAFM (x) merging results of tree crown detection on sparse forest test set.

**Figure 14:**
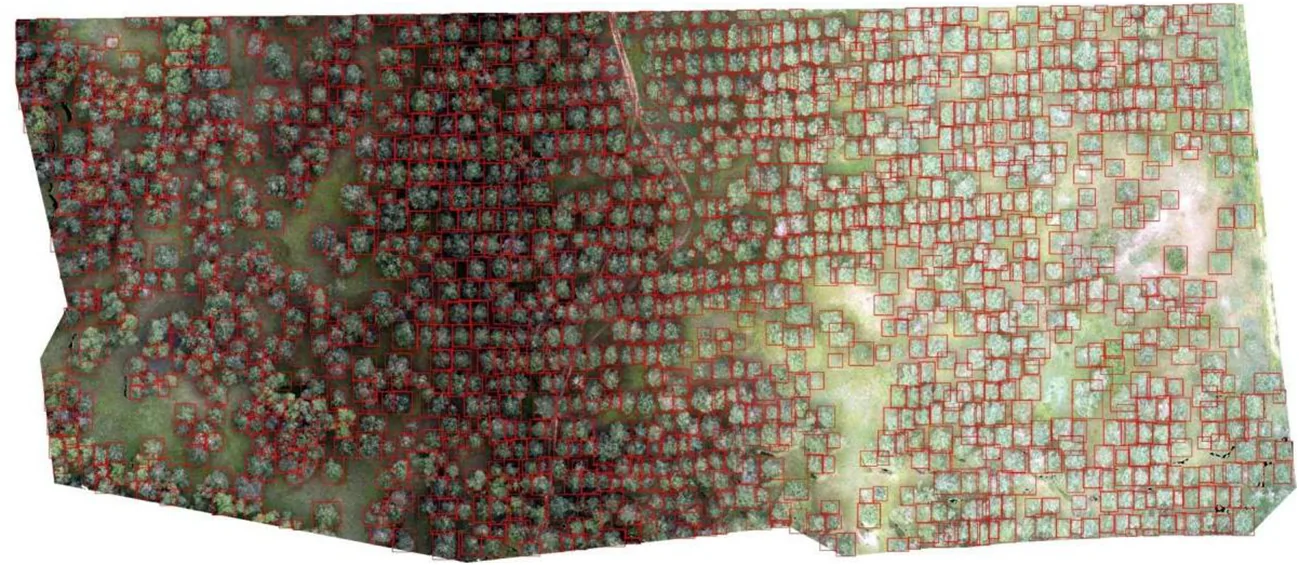
YOLOv8-CAFM (x) merging results of tree crown detection on dense forest test set.

Based on the above merged results, further regression evaluation was conducted between the individual tree detection results of the YOLOv8-CAFM model on the test set and the real sample label area, as shown in **Figure 15**. The accuracy of crown area detection in sparse and dense forests was R^2^ = 0.898, 0.794, and rRMSE=13.44% and 21.61%, respectively. The experimental results show that the YOLOv8-CAFM model exhibits a high linear correlation between the predicted values of tree crown area and the true values in both sparse and dense forest scenarios, indicating that the model can accurately extract individual tree crown boundaries in different scenarios. The sparse forest accuracy is higher than the dense forest, which may be attributed to the fact that the trees in the sparse forest are more dispersed and there is less shading between the canopies, allowing the model to capture the individual tree crowns more clearly.

**Figure 15:**
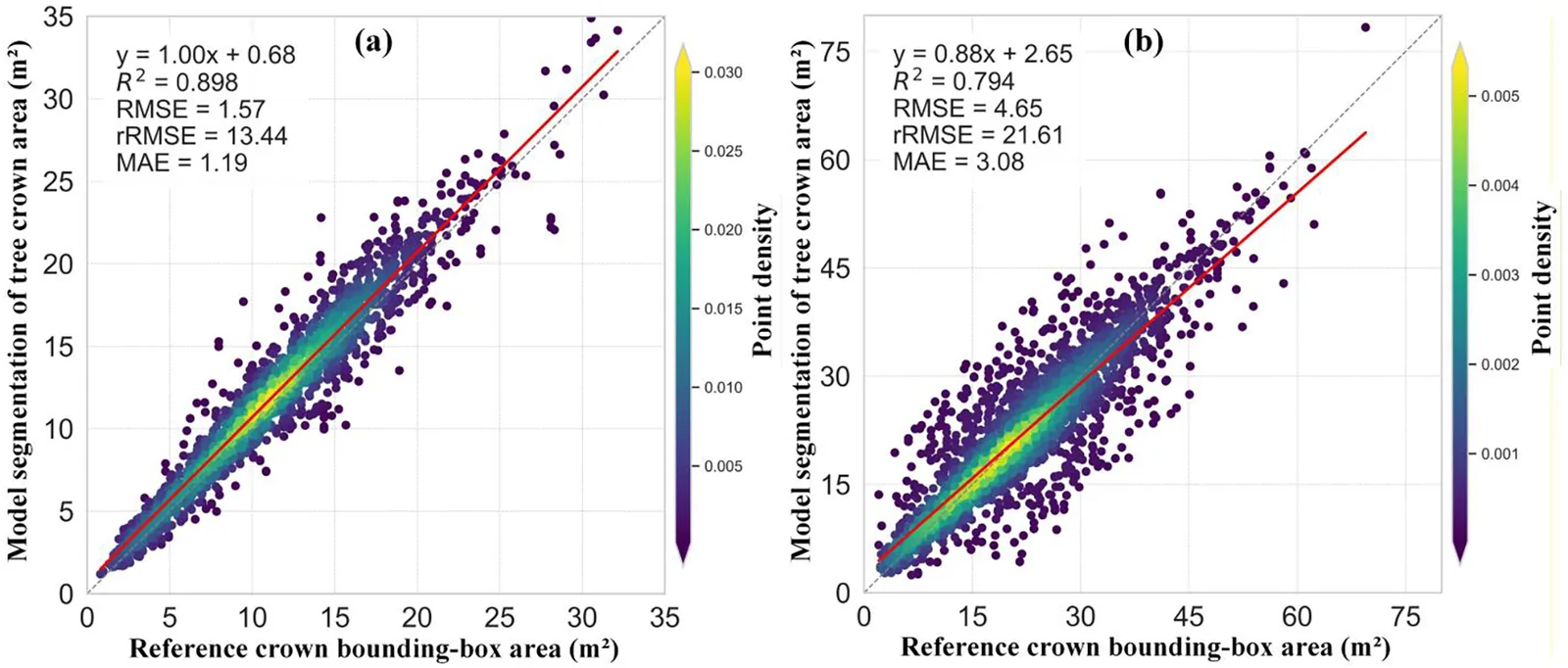
Evaluation of individual tree crown detection area of YOLOv8-CAFM in different forests. **(a)** sparse forest; **(b)** dense forest.

### 3.5 Mapping and accuracy assessment of AGC stocks in forests at the individual tree scale

Based on individual tree crown segmentation, different tree species’ allometric equations and carbon content were used to estimate the AGC stocks of individual tree scale forests in the study area (**Table 3**). The main tree species in the dense forest research area are *Pinus sylvestris* var. *mongolica* and *Pinus tabuliformis*, and the distribution areas of the two species have clear boundaries, so the species differentiation was directly carried out by visual interpretation. Firstly, a linear model between individual tree crown and DBH was constructed based on field-measured data (**Figure 16**), then DBH was calculated indirectly by using the crown area obtained from the UAV, and finally the individual tree scale AGC stock estimation results obtained from the tree height data in combination with allometric equations and carbon content rate (**Figure 17**). The experimental results showed that the estimated forest AGC stocks R^2^ for *Pinus sylvestris* var. *mongolica* and *Pinus tabuliformis* were 0.53 and 0.60, respectively, and the RMSE (%) were 16.40% and 31.61%, respectively.

**Figure 16:**
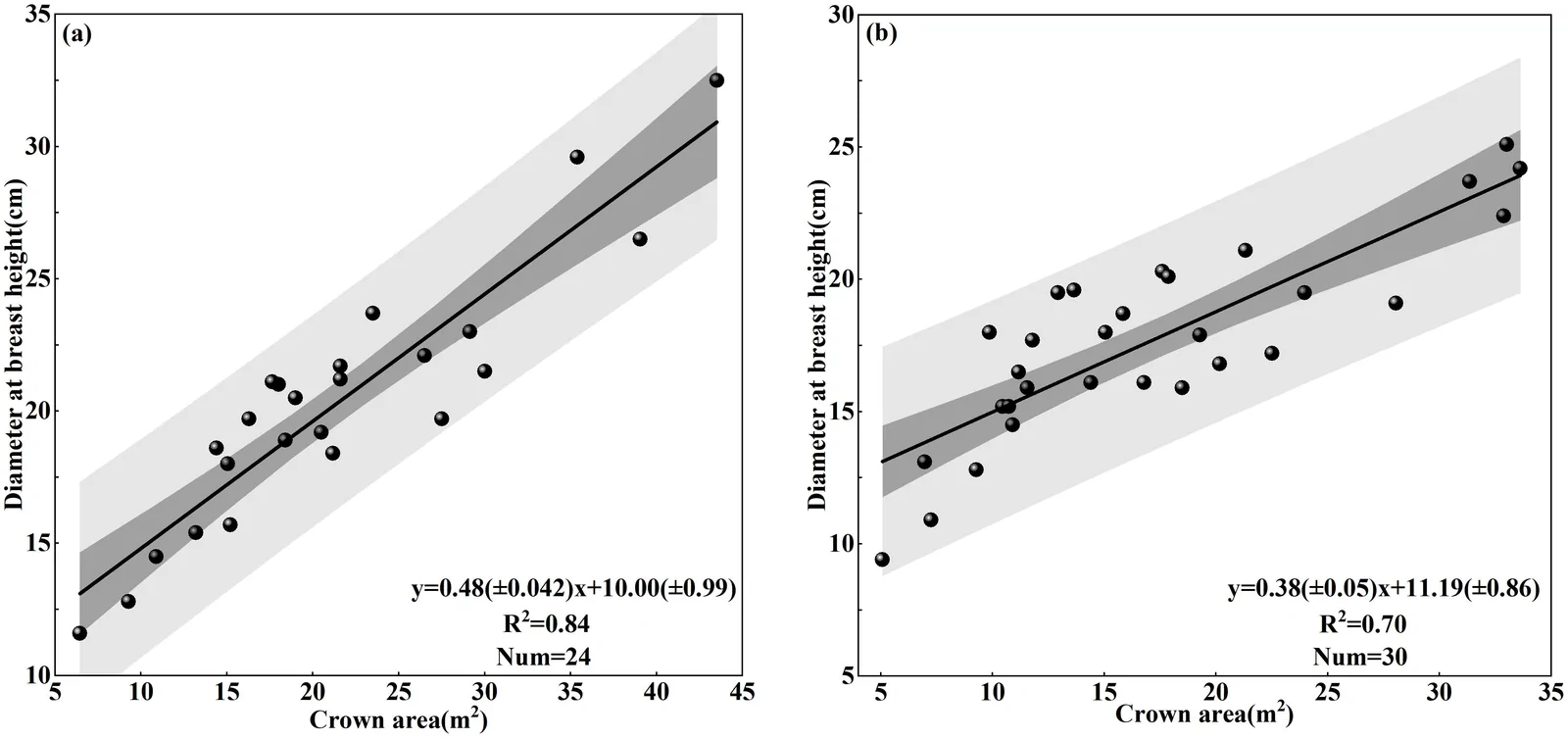
Results of the univariate linear regression model for DBH and crown area for different tree species. **(a)** *Pinus sylvestris* var. *mongolica*; **(b)** *Pinus tabuliformis*.

**Figure 17:**
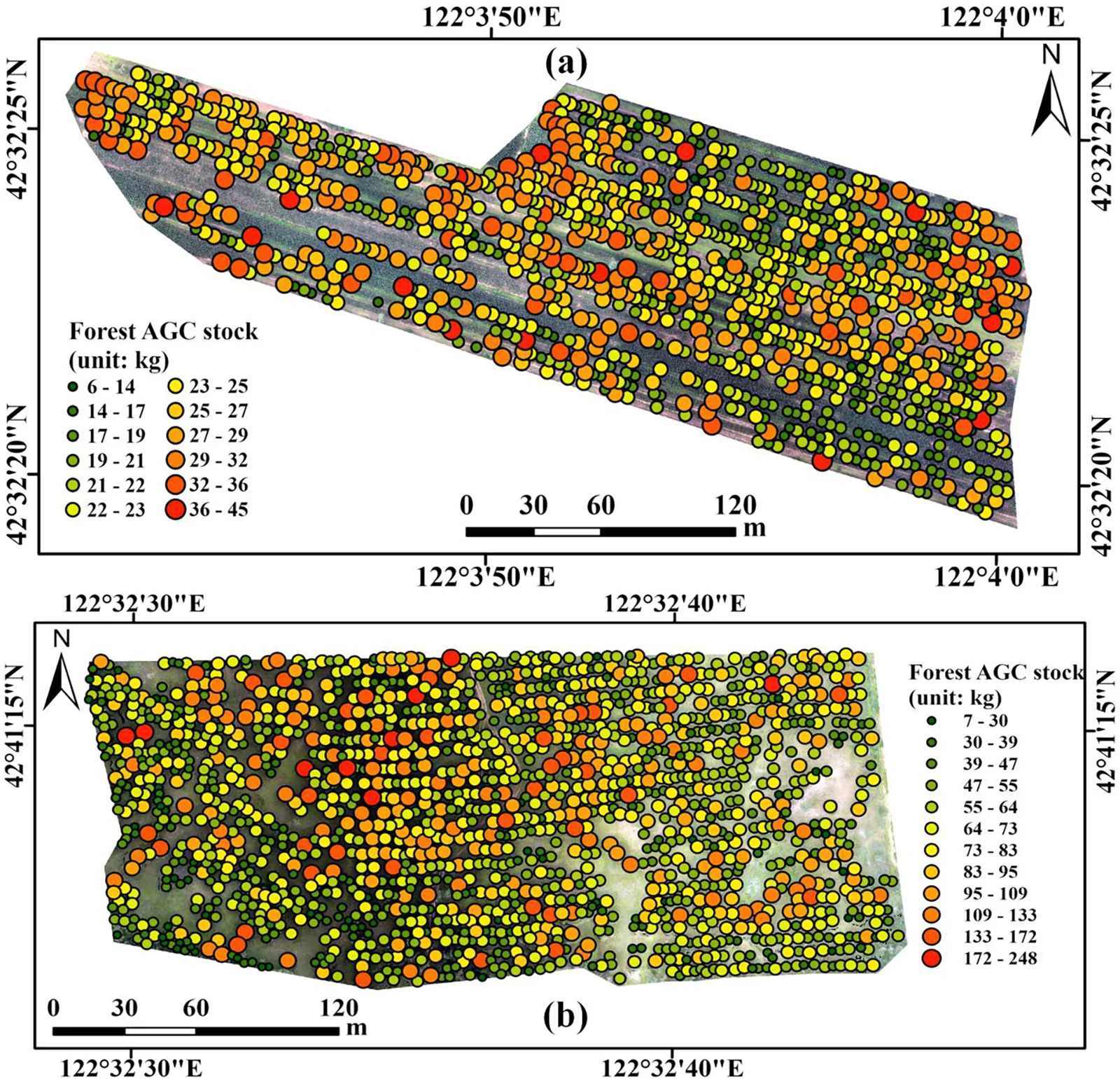
AGC stock estimation results of individual tree scales in different forest areas. **(a)** sparse forest; **(b)** dense forest.

## 4 Discussion

### 4.1 Canopy-dependent performance and the role of local-global feature fusion

The benefit of CAFM was strongly dependent on canopy condition. Across the five model scales, the mean absolute improvement in mAP@0.50:0.95 was 0.012 in the sparse-canopy stand but 0.069 in the dense-canopy stand. For YOLOv8-x, the corresponding improvements were 0.015 and 0.068. In the sparse-canopy stand, mAP@0.50 was already 98.7% for both YOLOv8-x and YOLOv8-CAFM-x, suggesting a ceiling effect for basic crown-presence detection. The larger improvement at stricter IoU thresholds and under dense-canopy conditions is consistent with the intended role of CAFM. The convolution branch retains fine-scale crown texture and edge cues, whereas the attention branch strengthens longer-range feature interaction and contextual representation. Their fusion may therefore be most useful when local crown cues are incomplete or ambiguous because of overlap, shadow, or similar appearance among neighboring crowns. This canopy-dependent response is ecologically relevant because increasing canopy closure and crown interaction alter both the visibility of individual crowns and the structural meaning of crown size. Improved crown localization can extend field observations of tree-level canopy organization and support crown-based analyses of structural variation and AGC. The comparison between the original YOLOv8 and the complete CAFM variant demonstrates an architecture-level performance gain, but it does not independently isolate the contribution of the local and attention branches or directly visualize the learned attention patterns. Dense-canopy performance also remained lower than sparse-canopy performance. Crown overlap, partial occlusion, and shadow may have contributed to this difference by weakening crown-boundary information and increasing visual similarity among adjacent trees. However, the two sites also differed in dominant species, image-acquisition year and date, illumination conditions, and stand structure. These factors were confounded with site identity in the present study and their individual effects could not be separated. Therefore, the poorer performance at the dense-canopy site should be interpreted as the result of the combined site and acquisition conditions rather than as an independent effect of canopy density or crown overlap alone. Thus, CAFM alleviated, rather than eliminated, errors caused by severe overlap and weak crown boundaries.

Although the YOLOv8-CAFM (x) model performs well in individual tree detection tasks, there are still some limitations in practical applications, for example, there is still room for improving the accuracy of the model in extremely dense forest scenes, so multi-scale feature fusion and object occlusion processing strategies can be further explored in subsequent studies, or multi-modal data fusion (such as thermal infrared images) can be introduced to further improve the performance of the model in complex scenes, and semi-supervised learning or self-supervised learning methods can also be combined to reduce the reliance on large amounts of labeled data.

### 4.2 Influence and limitations of the selected spatial resolution

Spatial resolution is an important factor affecting individual-tree crown detection from UAV imagery. In this study, imagery from both study sites was resampled to a common working spatial resolution of 0.06 m before image tiling and model analysis. This resolution was adopted to standardize the spatial characteristics of the model inputs across the two study sites. However, because the present study did not systematically compare multiple spatial resolutions, the selected resolution should not be interpreted as an experimentally determined optimal resolution. However, higher resolution is not necessarily better, when the resolution exceeds a certain threshold, its effect on improving model accuracy is limited, and may even lead to a decrease in model detection accuracy due to local detail interference. For example, Yin and Wang (2019) pointed out that the spatial resolution of the image should be at least one-fourth of the diameter of the tree crown. Compared with resolutions of 0.10 m, 0.50 m, and 1 m, a resolution of 0.25 m is the best choice for detecting an individual mangrove tree crown. Moreover, when the image resolution is 0.1 m, the accuracy is lower due to noise interference. Hao et al. (2023) first proposed the concept of crown resolution, and the results showed that when the crown resolution is in the range of 800–12800 pixels/tree, the crown detection accuracy is the highest, and suggested that the appropriate resolution should be selected according to the size of the tree crown in the practical application.

In principle, finer spatial resolution can preserve more detailed information on crown edges, small crowns, canopy gaps, and the spatial separation between adjacent trees, which may facilitate the detection of small or partially occluded crowns. However, finer imagery can also increase within-crown textural heterogeneity, shadow detail, background complexity, data volume, and computational cost. Conversely, coarser imagery may reduce local noise and computational requirements, but excessive spatial aggregation can blur crown boundaries and decrease the detectability of small or closely spaced trees. Therefore, the appropriate spatial resolution represents a trade-off among crown-level information preservation, background complexity, model performance, and computational efficiency. The 0.06-m imagery retained sufficient crown-level detail for applying the proposed detection framework under the conditions examined in this study. Nevertheless, the lower detection performance in the dense-canopy stand indicates that spatial resolution alone cannot fully resolve the effects of crown overlap, occlusion, shadow interference, and weak inter-crown boundaries. Spatial resolution should therefore be considered together with canopy structure and model feature-representation capability.

A limitation of this study is the absence of a systematic spatial-resolution sensitivity analysis. Consequently, the effects of resolution on crown-detection accuracy and downstream AGC estimation could not be quantitatively separated from those of stand structure and model architecture. Future studies should compare multiple resolutions derived from the same source imagery under consistent data partitions, training settings, and evaluation criteria. Such analyses should jointly evaluate detection accuracy, crown-size-dependent errors, computational efficiency, and the propagation of uncertainty to DBH and AGC estimates.

### 4.3 Assessment of AGC stock in forest plots

The allometric equation is a classic method for estimating forest biomass by establishing empirical equations between forest parameters such as tree height, crown width, DBH and biomass to estimate forest biomass, and then calculating carbon stocks in individual trees based on the carbon content of different species. This method has high accuracy and does not require deforestation, which can significantly reduce destructive sampling of forests (Navarro et al., 2020). In this study, due to the relatively single tree species in the study area, the species information was obtained through field survey and the relationship between crown area and DBH was established, so the individual tree scale carbon stock was estimated directly based on the results of canopy segmentation of UAV images and CHM, which relied on anthropogenic experience to a certain extent.

From **Figure 18**, it can be seen that the predicted AGC range of individual plants in the high canopy area is significantly underestimated, while low AGC trees are overestimated. The slope between the predicted value and the reference value is only 0.40, and the rRMSE is 31.61%. This deviation may be caused by multiple stages in the workflow. Crown overlap, shadowing, and partial occlusion may cause the visible or detected extent of large crowns to saturate or be truncated. Moreover, axis-aligned bounding-box area represents a crown-size proxy rather than the true crown polygon area and may compress the range of crown sizes. The linear crown area-DBH relationship can further shrink predictions toward the mean, particularly when the calibration sample contains relatively few trees at the upper end of the DBH distribution. Uncertainty in CHM derived height and in the photogrammetric DEM beneath the closed canopy is subsequently propagated through the species-specific allometric equation. Because AGC responds nonlinearly to DBH and height, these structural errors can have a disproportionately large effect on high AGC trees. Addressing this limitation will require larger calibration datasets covering the upper tail of the DBH distribution, crown-segmentation outputs rather than bounding-box proxies, evaluation of nonlinear or hierarchical crown-DBH relationships, and independent validation of terrain and canopy-height products.

**Figure 18:**
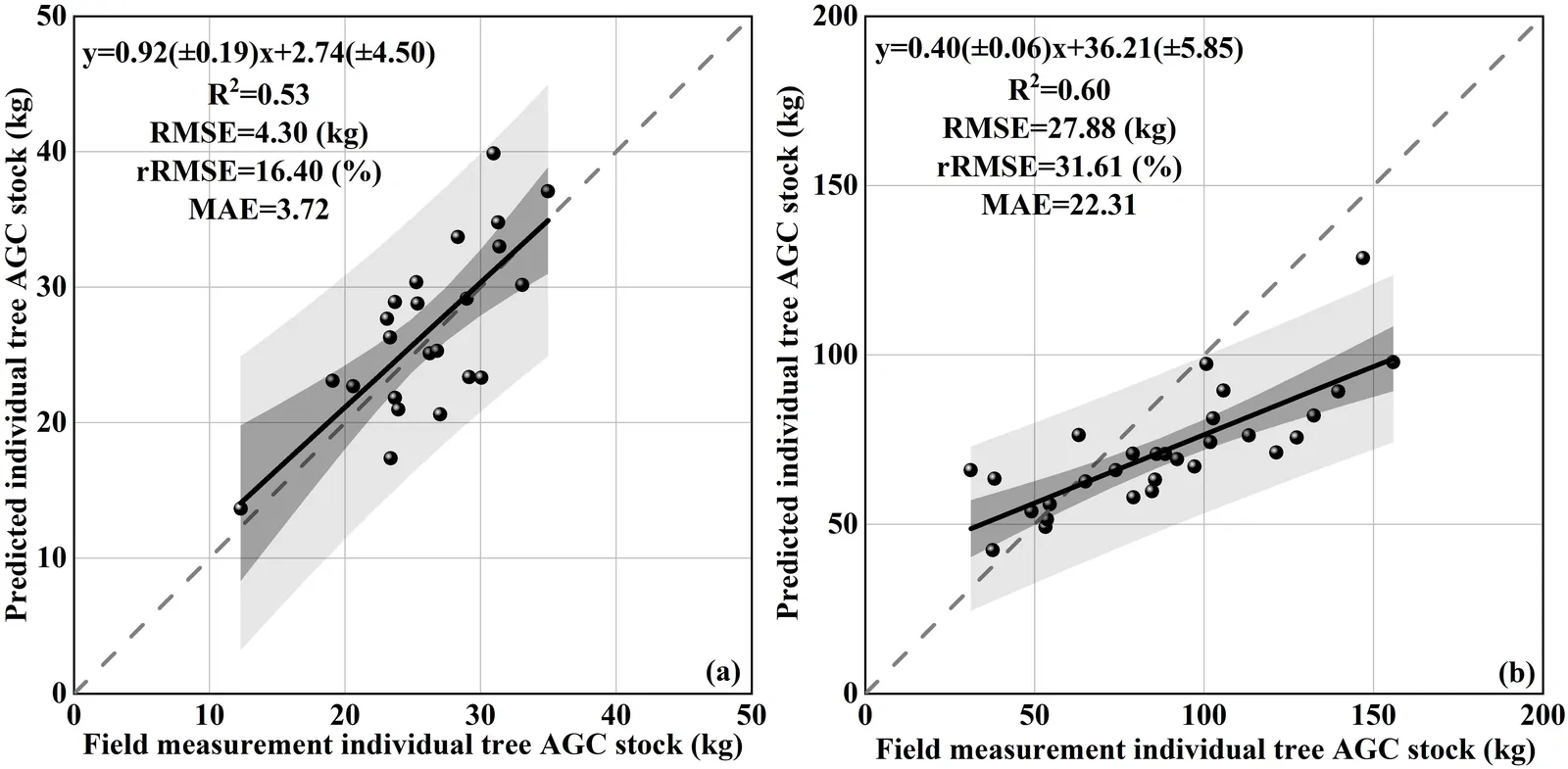
Scatter plot of AGC stock estimation result and field measurement for individual trees in different plots. **(a)** sparse forest; **(b)** dense forest.

Uncertainty in CHM derived height also contributes to the final estimates. However, the available results do not indicate that height uncertainty exceeds the influence of the crown area to DBH pathway. The published allometric equations and fixed carbon fractions introduce an additional source of structural uncertainty, including parameter error, residual variation, and possible limitations in their transferability to local stand conditions (Molto et al., 2013). Because the same equations and carbon fractions were used to calculate both reference and UAV-derived AGC, their uncertainty could not be independently evaluated in this study. Therefore, the identified dominant component refers to the observable remote-sensing pathway rather than the total uncertainty in true carbon stock.

## 5 Conclusion

This study focuses on an individual tree detection method based on deep learning and explores the performance of different object detection models in different forest density scenarios. Based on this, the AGC stock of individual tree scale was estimated. Firstly, an improved YOLOv8-CAFM model was proposed to address issues such as differences in canopy density, changes in light and shadow, and the complexity of tree crown background in forest environments. By introducing CNNs and attention fusion modules, the model significantly improved its detection accuracy in complex scenes. The experimental results showed that YOLOv8-CAFM (x) performs well in both sparse and dense forest scenes, and its mAP@0.50:0.95 is 0.736 and 0.609, with F1 scores of 0.974 and 0.856 respectively, which are significantly better than the compared models such as YOLOv8, Faster R-CNN, YOLOx, and Deepforest. Based on individual tree crown detection, the AGC stock of individual tree scale was estimated by combining different tree species’ allometric equations and carbon content, with the mean values in sparse forests and dense forests were 23.49 kg and 66.8 kg, respectively. The accuracy was verified based on field measurement data of *Pinus sylvestris* var. *mongolica* and *Pinus tabuliformis*, with R^2^ being 0.53 and 0.60, and RMSE (%) being 16.40% and 31.61%, respectively. The improved deep learning model significantly improves the efficiency of tree crown identification, which provides reliable technical support for forest resource surveys and ecological monitoring, and the results can provide a data basis for the estimation of AGC stock at the sample and regional scales.

## Data Availability

The raw data supporting the conclusions of this article will be made available by the authors, without undue reservation.

## References

[B1] Ball J. G. Hickman S. H. Jackson T. D. Koay X. J. Hirst J. Jay W. . (2023). Accurate delineation of individual tree crowns in tropical forests from aerial RGB imagery using Mask R‐CNN. Remote Sens. Ecol. Conserv. 9, 641–655. doi: 10.1101/2022.07.10.49948038621210

[B2] Brandt M. Tucker C. J. Kariryaa A. Rasmussen K. Abel C. Small J. . (2020). An unexpectedly large count of trees in the West African Sahara and Sahel. Nature 587, 78–82. doi: 10.1038/s41586-020-2824-5 33057199

[B3] Brandtberg T. Walter F. (1998). Automated delineation of individual tree crowns in high spatial resolution aerial images by multiple-scale analysis. Mach. Vision Appl. 11, 64–73. doi: 10.1007/s00138005009130311153

[B4] Buckland M. Gey F. (1994). The relationship between recall and precision. J. Am. Soc. For. Inf. Sci. 45, 12–19. doi: 10.1002/(sici)1097-4571(199401)45:1<12::aid-asi2>3.0.co;2-l

[B5] Cai C. Xu H. Chen S. Yang L. Weng Y. Huang S. . (2023). Tree recognition and crown width extraction based on novel Faster-RCNN in a dense loblolly pine environment. Forests 14, 863. doi: 10.3390/f1405086330654563

[B6] Cessna J. Alonzo M. G. Foster A. C. Cook B. D. (2021). Mapping boreal forest spruce beetle health status at the individual crown scale using fused spectral and structural data. Forests 12, 1145. doi: 10.3390/f1209114530654563

[B7] De Frenne P. Lenoir J. Luoto M. Scheffers B. R. Zellweger F. Aalto J. . (2021). Forest microclimates and climate change: Importance, drivers and future research agenda. Global Change Biol. 27, 2279–2297. doi: 10.1111/gcb.15569 33725415

[B8] Demissie W. A. Roccella S. Rossetto R. Minnocci A. Vannini A. Sebastiani L. (2025). “ Comparative analysis of deep learning models for olive tree crown and shadow segmentation towards biovolume estimation”, in: 2025 IEEE International Workshop on Metrology for Agriculture and Forestry (MetroAgriFor) (Bologna: IEEE), 303–308. doi: 10.1109/MetroAgriFor66923.2025.11512382

[B9] Duarte A. Borralho N. Cabral P. Caetano M. (2022). Recent advances in forest insect pests and diseases monitoring using UAV-based data: A systematic review. Forests 13, 911. doi: 10.3390/f1306091130654563

[B10] Fassnacht F. E. Latifi H. Stereńczak K. Modzelewska A. Lefsky M. Waser L. T. . (2016). Review of studies on tree species classification from remotely sensed data. Remote Sens. Environ. 186, 64–87. doi: 10.1016/j.rse.2016.08.01342574925

[B11] Ferreira M. P. Wagner F. H. Aragão L. E. Shimabukuro Y. E. de Souza Filho C. R. (2019). Tree species classification in tropical forests using visible to shortwave infrared WorldView-3 images and texture analysis. ISPRS J. Photogramm. Remote Sens. 149, 119–131. doi: 10.1016/j.isprsjprs.2019.01.01942574925

[B12] Gering L. R. May D. M. (1995). The relationship of diameter at breast height and crown diameter for four species groups in Hardin County, Tennessee. South. J. Appl. For. 19, 177–181. doi: 10.1093/sjaf/19.4.177

[B13] Gschwantner T. Alberdi I. Bauwens S. Bender S. Borota D. Bosela M. . (2022). Growing stock monitoring by European National Forest Inventories: Historical origins, current methods and harmonisation. For. Ecol. Manage. 505, 119868. doi: 10.1016/j.foreco.2021.11986842574925

[B14] Hao Z. Lin L. Post C. J. Mikhailova E. A. Li M. Chen Y. . (2021). Automated tree-crown and height detection in a young forest plantation using mask region-based convolutional neural network (Mask R-CNN). ISPRS J. Photogramm. Remote Sens. 178, 112–123. doi: 10.1016/j.isprsjprs.2021.06.00342574925

[B15] Hao Z. Lin L. Post C. J. Mikhailova E. A. Yu K. Fang H. . (2023). The co-effect of image resolution and crown size on deep learning for individual tree detection and delineation. Int. J. Digital Earth 16, 3753–3771. doi: 10.1080/17538947.2023.225763637339054

[B16] Hartling S. Sagan V. Maimaitijiang M. (2021). Urban tree species classification using UAV-based multi-sensor data fusion and machine learning. GIScience Remote Sens. 58, 1250–1275. doi: 10.1080/15481603.2021.197427537339054

[B17] Hodson T. O. (2022). Root mean square error (RMSE) or mean absolute error (MAE): When to use them or not. Geosci. Model. Dev. Discuss. 2022, 1–10. doi: 10.5194/gmd-2022-64

[B18] Hu G. Wang T. Wan M. Bao W. Zeng W. (2022). UAV remote sensing monitoring of pine forest diseases based on improved Mask R-CNN. Int. J. Remote Sens. 43, 1274–1305. doi: 10.1080/01431161.2022.203245537339054

[B19] Hu S. Gao F. Zhou X. Dong J. Du Q. (2024). Hybrid convolutional and attention network for hyperspectral image denoising. IEEE Geosci. Remote Sens. Lett. 21, 1–5. doi: 10.1109/lgrs.2024.337029925079929

[B20] Huang H. Li X. Chen C. (2018). Individual tree crown detection and delineation from very-high-resolution UAV images based on bias field and marker-controlled watershed segmentation algorithms. IEEE J. Sel. Top. Appl. Earth Obs. Remote Sens. 11, 2253–2262. doi: 10.1109/jstars.2018.283041025079929

[B21] Huang H. Xu H. Wang X. Silamu W. (2015). Maximum F1-score discriminative training criterion for automatic mispronunciation detection. IEEE/ACM Transactions on Audio, Speech, and Language Processing 23, 787–797.

[B22] Huo L. Lindberg E. (2020). Individual tree detection using template matching of multiple rasters derived from multispectral airborne laser scanning data. Int. J. Remote Sens. 41, 9525–9544. doi: 10.1080/01431161.2020.180012737339054

[B23] Imangholiloo M. Saarinen N. Markelin L. Rosnell T. Nasi R. Hakala T. . (2019). Characterizing seedling stands using leaf-off and leaf-on photogrammetric point clouds and hyperspectral imagery acquired from unmanned aerial vehicle. Forests 10, 415. doi: 10.3390/f1005041530654563

[B24] Kang Y. Jang E. Im J. Kwon C. (2022). A deep learning model using geostationary satellite data for forest fire detection with reduced detection latency. GIScience Remote Sens. 59, 2019–2035. doi: 10.1080/15481603.2022.214387237339054

[B25] Kapil R. Marvasti-Zadeh S. M. Erbilgin N. Ray N. (2024). “ Shadowsense: Unsupervised domain adaptation and feature fusion for shadow-agnostic tree crown detection from rgb-thermal drone imagery”, in: Proceedings of the IEEE/CVF Winter Conference on Applications of Computer Vision (WACV) (Waikoloa, HI), 8266–8276.

[B26] Kattenborn T. Leitloff J. Schiefer F. Hinz S. (2021). Review on convolutional neural networks (CNN) in vegetation remote sensing. ISPRS J. Photogramm. Remote Sens. 173, 24–49. doi: 10.1016/j.isprsjprs.2020.12.01042574925

[B27] Korznikov K. Kislov D. Petrenko T. Dzizyurova V. Doležal J. Krestov P. . (2023). Unveiling the potential of drone-borne optical imagery in forest ecology: A study on the recognition and mapping of two evergreen coniferous species. Remote Sens. 15, 4394. doi: 10.3390/rs1518439430654563

[B28] Lassalle G. Ferreira M. P. La Rosa L. E. C. de Souza Filho C. R. (2022). Deep learning-based individual tree crown delineation in mangrove forests using very-high-resolution satellite imagery. ISPRS J. Photogramm. Remote Sens. 189, 220–235. doi: 10.1016/j.isprsjprs.2022.05.00242574925

[B29] Ma Z. Pang Y. Wang D. Liang X. Chen B. Lu H. . (2020). Individual tree crown segmentation of a larch plantation using airborne laser scanning data based on region growing and canopy morphology features. Remote Sens. 12, 1078. doi: 10.3390/rs1207107830654563

[B30] Mielcarek M. Sterenczak K. Khosravipour A. (2018). Testing and evaluating different LiDAR-derived canopy height model generation methods for tree height estimation. Int. J. Appl. Earth Obs. Geoinf. 71, 132–143. doi: 10.1016/j.jag.2018.05.00242574925

[B31] Miraki M. Sohrabi H. Fatehi P. Kneubuehler M. (2021). Individual tree crown delineation from high-resolution UAV images in broadleaf forest. Ecol. Inf. 61, 101207. doi: 10.1016/j.ecoinf.2020.10120742574925

[B32] Molto Q. Rossi V. Blanc L. (2013). Error propagation in biomass estimation in tropical forests. Methods Ecol. Evol. 4, 175–183. doi: 10.1111/j.2041-210x.2012.00266.x40046247

[B33] Navarro A. Young M. Allan B. Carnell P. Macreadie P. Ierodiaconou D. (2020). The application of unmanned aerial vehicles (UAVs) to estimate above-ground biomass of mangrove ecosystems. Remote Sens. Environ. 242, 111747. doi: 10.1016/j.rse.2020.11174742574925

[B34] Nunes L. J. Meireles C. I. Pinto Gomes C. J. Almeida Ribeiro N. M. (2020). Forest contribution to climate change mitigation: Management oriented to carbon capture and storage. Climate 8, 21. doi: 10.3390/cli802002130654563

[B35] Onishi M. Ise T. (2021). Explainable identification and mapping of trees using UAV RGB image and deep learning. Sci. Rep. 11, 903. doi: 10.1038/s41598-020-79653-9 33441689 PMC7806907

[B36] Panagiotidis D. Abdollahnejad A. Surový P. Chiteculo V. (2017). Determining tree height and crown diameter from high-resolution UAV imagery. Int. J. Remote Sens. 38, 2392–2410. doi: 10.1080/01431161.2016.126402837339054

[B37] Pearse G. D. Tan A. Y. S. Watt M. S. Franz M. O. Dash J. P. (2020). Detecting and mapping tree seedlings in UAV imagery using convolutional neural networks and field-verified data. ISPRS J. Photogramm. Remote Sens. 168, 156–169. doi: 10.1016/j.isprsjprs.2020.08.00542574925

[B38] Puliti S. Astrup R. (2022). Automatic detection of snow breakage at single tree level using YOLOv5 applied to UAV imagery. Int. J. Appl. Earth Obs. Geoinf. 112, 102946. doi: 10.1016/j.jag.2022.10294642574925

[B39] Roslan Z. Long Z. A. Ismail R. (2021). “ Individual tree crown detection using GAN and RetinaNet on tropical forest”, in: 2021 15th International Conference on Ubiquitous Information Management and Communication (IMCOM) (Seoul: IEEE), 1–7. doi: 10.1109/IMCOM51814.2021.9377360

[B40] Safonova A. Guirado E. Maglinets Y. Alcaraz-Segura D. Tabik S. (2021). Olive tree biovolume from UAV multi-resolution image segmentation with mask R-CNN. Sensors 21, 1617. doi: 10.3390/s21051617 33668984 PMC7956790

[B41] Sun W. Liu X. (2020). Review on carbon storage estimation of forest ecosystem and applications in China. For. Ecosyst. 7, 1–14. doi: 10.1186/s40663-019-0210-238164791

[B42] Sun S. Mo B. Xu J. Li D. Zhao J. Han S. (2024). Multi-YOLOv8: An infrared moving small object detection model based on YOLOv8 for air vehicle. Neurocomputing 588, 127685. doi: 10.1016/j.neucom.2024.12768542574925

[B43] Terven J. Córdova-Esparza D.-M. Romero-González J.-A. (2023). A comprehensive review of yolo architectures in computer vision: From yolov1 to yolov8 and yolo-nas. Mach. Learn. Knowl. Extr. 5, 1680–1716. doi: 10.3390/make504008330654563

[B44] Tong F. Tong H. Mishra R. Zhang Y. (2021). Delineation of individual tree crowns using high spatial resolution multispectral WorldView-3 satellite imagery. IEEE J. Sel. Top. Appl. Earth Obs. Remote Sens. 14, 7751–7761. doi: 10.1109/jstars.2021.310074825079929

[B45] Van Coillie F. M. B. Devriendt F. R. Verbeke L. P. C. De Wulf R. R. (2013). Directional local filtering for stand density estimation in closed forest canopies using VHR optical and LiDAR data. IEEE Geosci. Remote Sens. Lett. 10, 913–917. doi: 10.1109/lgrs.2013.224204425079929

[B46] Wang J. Chen X. Cao L. An F. Chen B. Xue L. . (2019). Individual rubber tree segmentation based on ground-based LiDAR data and faster R-CNN of deep learning. Forests 10, 793. doi: 10.3390/f1009079330654563

[B47] Wang Y.-H. Su W.-H. (2022). Convolutional neural networks in computer vision for grain crop phenotyping: A review. Agronomy-Basel 12, 2659. doi: 10.3390/agronomy1211265930654563

[B48] Weinstein B. G. Marconi S. Aubry‐Kientz M. Vincent G. Senyondo H. White E. P. (2020). DeepForest: A Python package for RGB deep learning tree crown delineation. Methods Ecol. Evol. 11, 1743–1751. doi: 10.1101/2020.07.07.19155138621210

[B49] Wu J. Yang G. Yang H. Zhu Y. Li Z. Lei L. . (2020). Extracting apple tree crown information from remote imagery using deep learning. Comput. Electron. Agric. 174, 105504. doi: 10.1016/j.compag.2020.10550442574925

[B50] Xia J. Wang Y. Dong P. He S. Zhao F. Luan G. (2022). Object-oriented canopy gap extraction from UAV images based on edge enhancement. Remote Sens. 14, 4762. doi: 10.3390/rs1419476230654563

[B51] Yao N. Ni F. Wu M. Wang H. Li G. Sung W.-K. (2022). Deep learning-based segmentation of peach diseases using convolutional neural network. Front. Plant Sci. 13. doi: 10.3389/fpls.2022.876357 35693175 PMC9174939

[B52] Yin D. Wang L. (2019). Individual mangrove tree measurement using UAV-based LiDAR data: Possibilities and challenges. Remote Sens. Environ. 223, 34–49. doi: 10.1016/j.rse.2018.12.03442574925

[B53] Zhang Y. Zhang W. Yu J. He L. Chen J. He Y. (2022). Complete and accurate holly fruits counting using YOLOX object detection. Comput. Electron. Agric. 198, 107062. doi: 10.1016/j.compag.2022.10706242574925

[B54] Zhen Z. Quackenbush L. J. Stehman S. V. Zhang L. (2015). Agent-based region growing for individual tree crown delineation from airborne laser scanning (ALS) data. Int. J. Remote Sens. 36, 1965–1993. doi: 10.1080/01431161.2015.103004337339054

