# Supplementary material for: Deep learning-based automatic tree-level crown detection from high-resolution UAV imagery for aboveground carbon stock estimation
Source: Front Plant Sci. 2026 Sep 23;17:1958828. doi: 10.3389/fpls.2026.1958828 (PMC13642801; doi:10.3389/fpls.2026.1958828)
Supplement: Supplementary file 1 [file Table1.docx]

Supplementary Material

**Table S1. Point cloud data processing process information**

| Item | Information |
| --- | --- |
| Densified point cloud | about 872 pt/m^2^ |
| DSM filtering | Inverse Distance Weighting |
| Ground-point classification | Supervised machine-learning classification based on multiscale geometric and color features |
| DEM generation | Classification-guided smoothing and interpolation implemented in Pix4Dmapper |

Original data link: https://pan.quark.cn/s/43a7d1d49871
